# A variational-autoencoder approach to solve the hidden profile task in hybrid human-machine teams

**DOI:** 10.1371/journal.pone.0272168

**Published:** 2022-08-02

**Authors:** Niccolo Pescetelli, Patrik Reichert, Alex Rutherford

**Affiliations:** 1 The Collective Intelligence Lab, New Jersey Institute of Technology, Newark, NJ, United States of America; 2 School of Business, Economics and Law at the University of Gothenburg, Gothenburg, Sweden; 3 Max Planck Institute for Human Development, Berlin, Germany; Ulm University, GERMANY

## Abstract

Algorithmic agents, popularly known as bots, have been accused of spreading misinformation online and supporting fringe views. Collectives are vulnerable to hidden-profile environments, where task-relevant information is unevenly distributed across individuals. To do well in this task, information aggregation must equally weigh minority and majority views against simple but inefficient majority-based decisions. In an experimental design, human volunteers working in teams of 10 were asked to solve a hidden-profile prediction task. We trained a variational auto-encoder (VAE) to learn people’s hidden information distribution by observing how people’s judgments correlated over time. A bot was designed to sample responses from the VAE latent embedding to selectively support opinions proportionally to their under-representation in the team. We show that the presence of a single bot (representing 10% of team members) can significantly increase the polarization between minority and majority opinions by making minority opinions less prone to social influence. Although the effects on hybrid team performance were small, the bot presence significantly influenced opinion dynamics and individual accuracy. These findings show that self-supervized machine learning techniques can be used to design algorithms that can sway opinion dynamics and group outcomes.

## 1 Introduction

The effect and influence of minority opinions on social groups have long been studied in psychology and sociology [1–4]. More recently, public interest in these questions has increased due to media coverage of troll factories, social bots and automated accounts on public fora [5, 6]. However, few studies have experimentally measured social bots’ ability to selectively support minority opinions and influence collective outcomes. In this paper, we trained a variational auto-encoder (VAE) to recognize shared information sources among a team of people solving a hidden profile task. We then used the VAE model to program a bot to selectively support minority opinions and quantified the effect on teams’ opinions and teams’ accuracy.

The presence of automated accounts, popularly known as social bots, is well documented on social media [7, 8]. Their goals and tasks can vary from innocuous chatbots to more malicious automated accounts [9]. Social media bots, in particular, have been accused of spreading hyper-partisan content to push political agendas and increase the perception of fringe views [10]. Bots may inflate the perception of particular views’ popularity and contribute to the spread of misinformation, conspiratory theories, and partisan messages [5, 11]. Whether bots can be intentionally designed to improve group performance is a matter of debate [12, 13]

Collectives, like groups, teams and online crowds, are vulnerable when information to solve a task is unequally distributed across multiple players [14, 15]. This scenario is known in psychology as the hidden profile problem. Some pieces of information are shared by a majority of players, while other pieces of information are known only by a minority of players (hidden information). The typical finding is that players tend to discuss and use information shared by the majority rather than the (still useful) information available only to minority players. We hypothesized that, in this scenario, artificially supporting minority opinions should improve team performance by rebalancing information distribution in the group. The hidden profile paradigm offers the opportunity to test the effect of a bot supporting minority views in a task where such influence can benefit group outcomes.

Threats (and opportunities) of deploying bots in social networks are consequential when people’s opinions are aggregated to form collective decisions like during democratic processes and elections or when opinions are aggregated to forecast future economic and geopolitical outcomes. For instance, researchers have documented bots’ potential for distorting information and exerting undue influence on popular opinions in real-world democratic decisions [5, 16–18]. These concerns have stimulated research on the subject [19–22] and have mobilized platforms to improve automatic detection and removal of algorithmic accounts [16, 23–25]. We contribute to this literature by advancing the hypothesis that, in addition to removing malicious bots, an equally effective strategy to combat misinformation is to design bots expressing views that increase opportunities for individuals to be exposed to hidden information.

In this paper, we experimentally study these phenomena in a modified hidden profile paradigm [14, 15]. We asked teams of volunteers to make weather predictions based on random indicators (humidity, temperature, and wind speed) with individual predictive patterns, which had to be learned from trial and error. Although outcomes were predicted by a linear combination of all indicator variables, each individual could observe only one indicator. Indicators were unequally distributed among the team, with one indicator shown to the majority of participants (majority group, 50%), while two more were shown to two minority groups (30% and 20% respectively). In each round, participants were asked to give an independent forecast and then revise it based on the forecasts made by others in their team.

Our research paradigm is motivated by two research programs. First, research in collective intelligence demonstrated that information independence is a crucial prerequisite for optimal information aggregation [26, 27]. Correlations between individuals—*e*.*g*., multiple people sharing the same piece of information in a hidden profile task—often lead to suboptimal outcomes [28, 29]. Second, advances in machine learning have improved both methods and the availability of clustering algorithms. These algorithms exploit correlations between individuals to reduce the feature space and find a low-dimensional information-preserving data representation. Given their formal similarity, we here apply a variational auto-encoder to the hidden profile problem.

We hypothesize that a bot selectively supporting minority views would help team performance by reducing the majority’s influence in this task. Instead of telling the bot whether people held majority or minority information sources, we trained a variational autoencoder (VAE) to learn this representation in an unsupervized fashion by observing the correlation of people’s responses over the first 110 rounds of the game. VAE-based approaches are used in several domains to generate low-dimensional interpretable embeddings from complex data [30]. Using a VAE allowed us to generate original opinions by sampling from the hidden, latent representation layer of the VAE, rather than simply copying human opinions [25, 31]. Our bot used VAE’s representation of people’s similarities (based on past trials) to strategically position itself in the opinion space. In the last 50 rounds of the game, we replaced a human player in the review stage with the VAE bot. Bot’s responses were in between the average majority response and the minority response but closer to minority views. Thus the bot positioned itself in a region of opinions where people are more susceptible to social influence, sometimes referred to as ‘latitude of acceptance’ [4]. This strategy may be more flexible and adaptive than simple opinion copying because it is adaptive to opinion shifts in humans over time. While current bots may have a significant impact because of their sheer number, strategic adaptive bots may be more effective at influencing the social system they are plugged in.

Furthermore, we hypothesized that by adopting opinions between majority and minority views, the bot could bridge the gap between the two opinion clusters and facilitate consensus. We compare the effect of such an adaptive bot with a random bot [32]. We conducted a series of exploratory data analyses that we report below. Our findings suggest that instead of bridging the gap between majority and minority views, the VAE bot further increased the team’s polarisation by making minority views less prone to social influence by the majority. Reduced majority influence was especially true when facing challenging rounds; thus people were more likely to seek social information. The bot’s influence on team performance was relatively weak but followed a similar pattern. In minority groups, the VAE bot improved initial judgments.

## 2 Methods

### Procedure

The study was approved by the Ethics Committee of the Max Planck Institute for Human Development. The game was implemented in Empirica [33]. The game’s task consisted in predicting a binary weather outcome (Rain *vs*. No Rain) based on the information provided by independent weather indicators. Participants were tested in teams of ten. Within each team, participants were assigned to one of three weather conditions: “wind”, “temperature” or “humidity”. These experimental conditions determined which weather indicator a given participant could see during the game—*e*.*g*.participants in the “wind” group could see only the wind indicator on any given round. Our design was similar to the hidden profile paradigm [14]. First, the correct outcome could only be predicted by knowing and aggregating all weather indicators together. Second, each weather indicator was equally important, *i*.*e*., equally predictive of the outcome (see Eq 4 below). Third, some weather indicators were more readily available to the group than others. In particular, we manipulated the number of people accessing each weather indicator. The first weather indicator was given to five participants in a group (majority). The second indicator was given to three participants (mid-size minority), and the third one was given to two participants (small minority). Thus, a simple opinion average or simple voting would have resulted in the greater but suboptimal influence of the majority indicator. A group needs to recognize and aggregate independent information sources rather than individual opinions to succeed. We randomized which weather indicator was assigned to which indicator group size (majority, mid-size minority or small minority) across the thirty games we ran.

The training phase had 110 rounds. In each round, a participant could see a weather indicator (“stimuli”) on a percentage scale, after which she was asked to predict the chances of rain within a 10-second window. The prediction was provided on a probabilistic scale of 0 to 1, where one corresponded to being certain that it will rain and 0 corresponded to being certain that it will not rain. After each round, participants could see the outcome for 5 seconds, and the Brier score calculated from their predictions was added to their score total. We used Brier scores for its strictly proper scoring property to discourage over- and/or under-confidence. Brier scores were computed using the formula proposed in the original paper by Glenn Brier [34] (S1 Equation in S1 File).

In the interaction phase lasting for 50 rounds, participants continued to see measurements for their respective indicator categories (wind, temperature, humidity). However, their forecast was now shared with other participants in their team. After seeing each other’s forecasts, participants had 10 seconds to revise their initial guess and provide a final forecast for every round.

**Fig 1:**
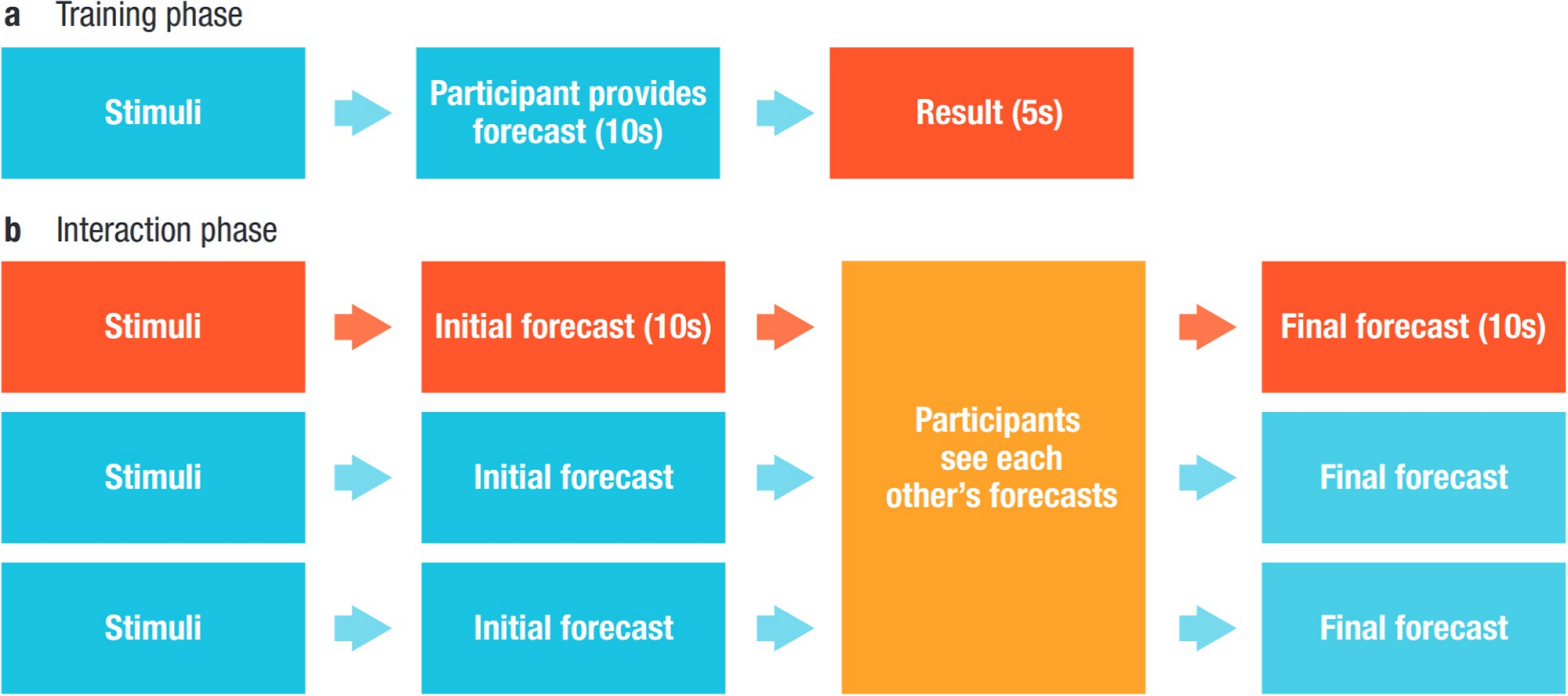
Experiment timeline. (a) During the first 110 rounds, participants see measurements from their indicator group and need to provide weather forecasts. On each round they receive feedback (“It rained” *vs*. “It did not rain”), thus allowing them to learn the predictive relation between the indicator and the outcome. (b) During the interaction phase, after seeing their indicator measurements and providing their independent answer, participants could see the forecasts made by other participants, before receiving round feedback.

During the interaction phase, we included an algorithmic player in each team. In 15 of the 30 teams (treatment teams), this algorithmic player provided forecasts based on the VAE model we trained on the respective team’s training phase data (Fig 1). In the other 15 teams (control teams), the algorithmic player provided randomly generated guesses. We compared the effect of including the VAE algorithm as an algorithmic player in a team on performance to a random baseline.

### Stimuli

The stimuli *x*_(⋅)_ for temperature, humidity and wind were randomly generated for all rounds of the game prior to its start. Values were drawn from a random uniform distribution in the interval [0, 1]. The values generated were then transformed into predictor values. The function used for this transformation was different for each predictor:

$$\begin{array}{c} \text{predictor}\;{\text{value}}^{\text{hum}}=1+x_{\text{hum}}+0.01\epsilon \end{array}$$ (1)

$$\begin{array}{c} \text{predictor}\;{\text{value}}^{\text{temp}}=1-x_{\text{temp}}+0.01\epsilon \end{array}$$ (2)

$$\begin{array}{c} \text{predictor}\;{\text{value}}^{\text{wind}}=\{\begin{array}{cc} 2, & x_{\text{wind}}\geq 2\\ 50x_{\text{wind}}^{2}-50x_{\text{wind}}+10+0.01\epsilon , & -2<x_{\text{wind}}<2,\\ -2, & x_{\text{wind}}\leq -2 \end{array} \end{array}$$ (3)

Where *ϵ* ∼ *N*(0, 1) is a random noise term. These functions (ignoring the random error component) led to a positive linear relationship in the case of humidity, a negative linear relationship for temperature, and a semi-quadratic relationship for wind (see S1 Fig). In other words, the chances of rain were highest for (1) high humidity values; (2) low-temperature values; and (3) low and high wind values. All three predictors were in the range [0, 1] (S1 Fig) and used arbitrary units. The first reason to use arbitrary units on the same scale rather than physical units was to keep error magnitudes similar (Kao et al. 2018; Fechner 1860). Furthermore, using arbitrary units limited the impact of participants’ prior knowledge on their outcome predictions.

Stimuli and the predictor values for all 160 rounds were generated before the game started for a given team. Taking these 160 values for each predictor category as the “sample”, we transformed the predictor values to z-scores. This transformation ensured that the different functions’ ranges (notice the different ranges on the y-axis in S1 Fig) converged onto a similar scale and that each predictor had the same weight on the outcome (Eq 4). Negative z-scores had a negative effect on rain probability, while positive z-scores increase the likelihood of rain.

We then summed up the three z-scores—one for wind, temperature, and humidity each—from a given round, and transformed the resulting sum using a sigmoid function to get our rain probability for round *k* (see S2 Fig):

$$\begin{array}{c} P\left({\text{rain}}_{k}=1|Z_{k}\right)=\frac{1}{1+\text{exp}(-5Z_{k}))} \end{array}$$ (4)

Where $Z_{k}\equiv z_{k}^{\text{temp}}+z_{k}^{\text{hum}}+z_{k}^{\text{wind}}$ for round *k*.

### Variational autoencoder

We used our two-stage experiment to train a variational autoencoder model (“VAE” in the following) on data collected directly from participants during the first stage of the experiment (“Training phase”). We then use the VAE as an algorithmic player in the second part of the experiment (“Interaction phase”). We compared behavior in groups where the algorithmic player followed the VAE with groups where the algorithmic player provided random responses.

**Fig 2:**
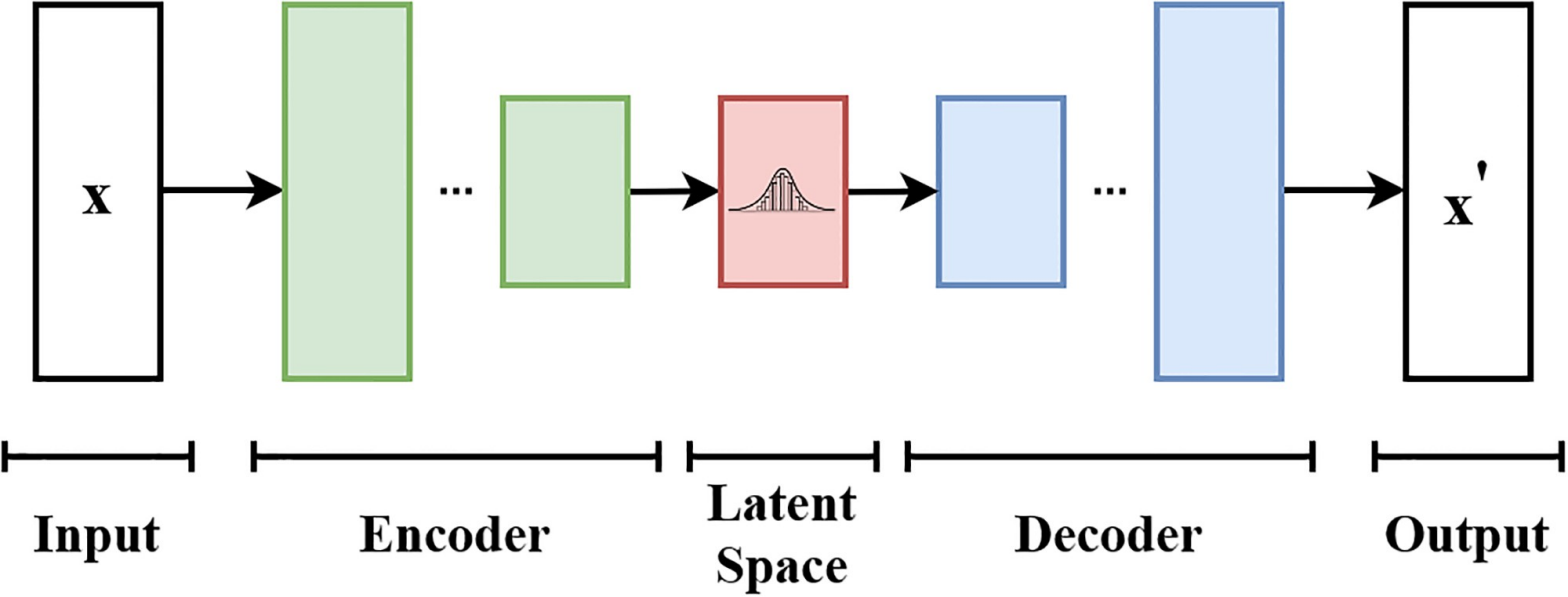
Schematic architecture of a variational auto-encoder. The input X is compressed by the encoder layer (green) into a hidden representational layer (red). A decoder (blue) takes as input the compressed representation and attempts to reproduce the original input as closely as possible (X’). Credit:EugenioTL, Wikipedia Commons contributor (CC BY-SA 4.0).

The VAE model was implemented using the Keras.js package and trained it on the first 110 rounds (Training phase) with a burn-in of 10 rounds to allow the players to familiarize with the task (Fig 2). An autoencoder is a convolutional neural network that converts a high-dimensional input into a low-dimensional one (the latent vector), and tries to reconstruct the original input with the highest quality possible from this vector. Additionally, the VAE includes a sampling step by which an input is encoded and used to parameterise a distribution from which the input into the deocder is sampled. Our VAE consisted of two connected networks, one encoder and one decoder. When training the model, the encoder took as input the response matrix *X* collected during the training phase. Each participant in the game was treated as one feature. Responses on each round were treated as different observations. The encoder returned a lower dimensionality vector than the initial input features. The low dimensional space was a hidden layer with three nodes. This procedure compresses redundant information from response correlations among participants, akin to other dimensionality reduction techniques such as principal component analysis (PCA). The VAE differs in that (i) the encoding and decoding process is, in general, non-linear and (ii) the hidden nodes learn a *distribution* over latent variables, and so their response is probabilistic. Although other techniques exist for modeling the non-linear relationships (*e*.*g*., SVMs), we used a VAE because it is a generative model that can be used to sample new unseen data from hidden representations in a systematic way. Contrary to other generative models (*e*.*g*., generative adversarial networks), VAEs’ representations are continuous and meaningful allowing to sample new data from a smooth latent space. The architecture of the VAE requires many hyper-parameters to be set and optimized typically. In this study, we aim to prove that a VAE can perform the function of a minority supporting bot rather than optimizing this behavior. The latent vector was then fed to the decoder trained to reconstruct the original data by minimizing the difference between the input matrix *X* and the output matrix *X*′. Compared to other dimensionality reduction techniques, such as principal component analysis, the advantage of this technique is that the latent space can be used to generate new data that has never been seen by the model. In our weather forecasting task, this results in a bot that is able to produce forecasts that were not made by any other human player. To train the model, we used default parameters in the Keras.js package.

After training, we proceeded in three steps to create an algorithmic agent that could operate in the second stage (“Interaction phase”): (1) we apply K-means (k = 3) to the last output layer’s weights; (2) we create a new set of coordinates along the hidden layer (corresponding to a new set of weights) by computing the weighted average of cluster centroids obtained in 1; (3) we replace the weights of the last output layer of the VAE with this new set of weights.

**Fig 3:**
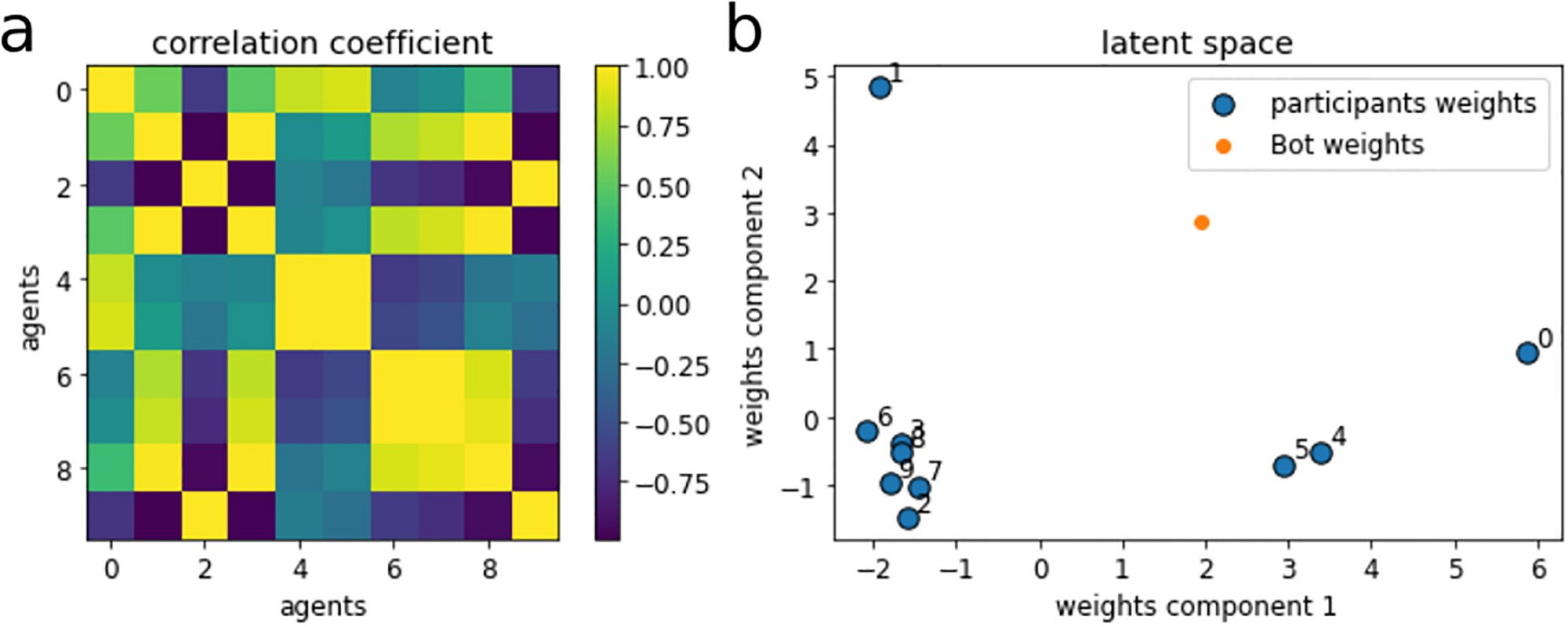
Example of a latent space of a variational autoencoder model trained on one of the teams in the study. (a) Correlation coefficient of the weights to the output layer of the variational autoencoder, after training. The clear structure of the data, as learned by the model, is seen in the clusters of high correlation coefficients. (b) The weights to the output layer projected on a 2-dimensional surface using principal component analysis. Three distinct clusters can be seen in the figure, each corresponding to a team of participants with similar response patterns. The model learned distinct representations corresponding to one large cluster (majority opinion) and two smaller clusters (minority opinions) as shown by the clear separation of the model’s weights in latent space. The bot player is represented in orange and shows a response pattern closer to the smaller clusters (minority opinions) than to the larger cluster (majority opinions).

1. We tested this procedure in a computer simulation using synthetic data before running the experiment. The code is publicly available on Open Science Framework, together with the rest of the analyses accompanying this paper. We show that the last layer’s weights correctly separate participants by condition. In other words, the participant distance along the hidden layer represents, after training, their response similarity across rounds. Fig 3 reproduces the same weights analysis with one of the teams tested. Fig 3a shows the correlation coefficients of the 10 sets of weights in the last layer of the model. High correlation coefficients represent greater weight similarity. The model learned to assign similar weights to participants giving similar responses. Similarly, Fig 3b shows a 2-dimensional projection of the output weights. Each blue point corresponds to one participant in a test team. A lower distance in the projected space corresponds to a similar pattern of weights from the hidden layer to the output layer of the VAE.
2. The algorithmic participant’s profile was specific for each team of participants and was defined as a new set of three coordinates along the hidden layer. The set of coordinates was obtained by averaging the cluster centroids, using weights **w** inversely proportional to the number of participants *N* in each cluster *c*:
  $$\begin{array}{c} w_{c}={\left(1/N_{c}\right)}^{\gamma } \end{array}$$ (5)
  where *γ* is the parameter controlling the bias towards supporting minority views (here set to 2). Fig 3b shows a 2-d projection of the set of weights that this procedure assigned to the algorithmic participant in one of the teams recruited (orange dot). Notice that the bot has a shorter distance (*i*.*e*. more similar weights) from the two smaller clusters (minority opinion clusters) than to the larger cluster (majority opinion cluster).
3. After defining the algorithmic player’s profile, we used it to sample a new response on each subsequent round (Interaction phase). To do that, we replaced at random one output unit of the VAE (corresponding to one human player) with our new set of weights representing the algorithmic player. We then run this new VAE model on all subsequent new (human) forecasts to obtain the responses of our algorithmic player. The net effect is that the VAE bot produces an original (unseen) forecast based on the pattern of forecasts produced by other human players.

We provide to the interested reader accompanying code on OSF (https://osf.io/pbc4v) showing the step-by-step procedure to generate a virtual player using synthetic data.

### Participants

The experiment was run double-blind on Prolific.co. We randomly allocated incoming participants between the control and treatment teams until we reached the desired 30 teams (15 control and 15 treatment). Participants gave informed consent via the experiment’s browser interface.

We paid participants £8 to complete the experiment that was estimated to take 55 minutes (equivalent to £8.57/hour). On top of the flat compensation rate, participants were ranked within their group of 10 based on their score achieved during the experiment. The participant with the highest score received an additional £10 and the participant ranked second received a bonus of £5. We discuss the potential downsides of tournament incentives later in the paper.

Our participant pool is diverse compared to most laboratory experiments using student subject pools, helping us to ease some of the concerns regarding the external validity of our findings. The summary statistics of our sample demographic information is presented in the S1 Table in S1 File. The mean and median age of participants is 31.8 years and 30 years, respectively (with a standard deviation of approximately 10 years). The sample is balanced with respect to gender, having 52.4% male participants.

Most participants (64.5%) reported English as their first language, and the most represented nationalities with respect to the total number of participants were the UK (38.8%) and the US (21.1%). 47.8% of participants were full-time employed, and students represented 30.8% of our sample.

## 3 Results

### Team polarization

We first tested whether the VAE bot helped reduce team polarization. Although the VAE player supported minority views more than majority views, it also represented a (weighted) average of each opinion cluster. Thus, we expected the VAE bot to bridge the gap between opinion clusters and increase alignment [29]. To test this hypothesis, we compared polarization in teams assigned to the VAE condition with polarization in control teams. We measured polarization as the standard deviation of forecasts [35], which we calculated for forecast distributions both before (*pre* SD) and after (*post* SD) exposure to peer information. Our measure of interest was ΔSD, defined as the change in the standard deviation of forecasts before and after peer exposure.

Participants saw different information according to which predictor group they were assigned to. Therefore, if one predictor suggested a high likelihood of rain while another the opposite, this would itself result in a higher standard deviation of forecasts. To control for these spurious effects, we measure *Z Range*, which calculates the distance between the largest and smallest z-score of the three predictor values in each round:

$$\begin{array}{c} ZRange_{j,k}=\text{max}\left(z_{j,k}^{\text{temp}},z_{j,k}^{\text{hum}},z_{j,k}^{\text{wind}}\right)-\text{min}\left(z_{j,k}^{\text{temp}},z_{j,k}^{\text{hum}},z_{j,k}^{\text{wind}}\right) \end{array}$$ (6)

For team *j* and round *k*. We ran two mixed-effects models with a random effect for teams (nested within the game’s date). The first model tested whether the change in polarization ΔSD was affected by our bot treatment (dummy coding: 1 = VAE bot, 0 = random bot), and controlling for z-score range. In the second model, the dependent variables was changed to post-interaction polarization (*post* SD). Results are shown in Table 1.

**Table 1:** Mixed models for team polarization. The presence of the VAE bot in hybrid teams increased the likelihood of polarization and the spread of forecasts post social interaction.

Dependent Variable:	*post* SD		ΔSD	
Predictors	Estimate	*p-value*	Estimate	*p-value*
(Intercept)	-0.18	0.169	-0.14’	0.086
Treatment	0.35’	0.052	0.28*	0.012
Z Range	0.11***	<0.001	-0.03	0.416
Random effects	Yes		Yes	
Observations	1500		776	
Marginal / Conditional R^2^	0.043 / 0.270		0.021 / 0.073	

Post-interaction polarization also tended to be higher in VAE condition compared to the control (*β* = .35, *p* = .052). As expected, a higher *Z Range* predicted increased polarization, but did not affect the change in polarization ΔSD. We thus focused on ΔSD as this was less affected by round-by-round fluctuations in the indicators’ range. The presence of VAE bots significantly increased polarization compared to a random bot baseline (*β* = .28, *p* = .01). These findings suggest that although the bot provided responses between minority and majority opinions, its bias to support minority opinions more than majority opinions led to an overall increase in polarization compared to the baseline.

### Influence of minority opinion

One hypothesis for the increased polarization observed in VAE condition was that, by supporting minority views, the VAE bot increased the influence of people holding those views against a majority. The presence of the VAE bot may have made people holding minority opinions less prone to social influence. If the minority were more influential than the control, one would expect that the final forecasts of majority groups would shift closer to the minority’s initial forecasts. We thus looked at whether final forecasts of the two largest sub-groups (the 5-person majority and the 3-person mid-size condition) in teams with the VAE bot were closer to initial minority forecasts. We calculated *Minority Influence* as the absolute difference between the 2-person minority’s median initial forecast and median final forecast of 3 and 5-person sub-groups separately, for each team *j* and round *k*:

$$\begin{array}{c} \text{Minority}\;{\text{Influence}}_{j,k}^{\text{majority}}=1-\left|\text{med}\left({\text{prediction}}_{I_{j,k}^{\text{minority}}}^{\text{initial}}\right)-\text{med}\left({\text{prediction}}_{I_{j,k}^{\text{majority}}}^{\text{final}}\right)\right| \end{array}$$ (7)

$$\begin{array}{c} \text{Minority}\;{\text{Influence}}_{j,k}^{\text{mid-size}}=1-\left|\text{med}\left({\text{prediction}}_{I_{j,k}^{\text{minority}}}^{\text{initial}}\right)-\text{med}\left({\text{prediction}}_{I_{j,k}^{\text{mid-size}}}^{\text{final}}\right)\right| \end{array}$$ (8)

Where $I_{j,k}^{\text{minority}}$, $I_{j,k}^{\text{mid-size}}$ and $I_{j,k}^{\text{majority}}$ denotes participants, who are assigned to the sub-group with a total of two, three and five participants, respectively. The variable is calculated for each team *j* and for each round *k*. A greater value indicates a greater influence of the minority cluster.

We control for round difficulty for 3 and 5-person sub-groups, as any outside sub-group influence is likely to be diminished in easy rounds (see Supporting information for a calculation of round difficulty). We calculate *Round Difficulty* depending on which predictor category (temperature, humidity, or wind) was assigned to the majority condition (N = 5) and the mid-size condition (N = 3). This was done to consider that, for instance, a linear relationship (*e*.*g*. temperature) was easier to learn than a quadratic one (*e*.*g*. wind).

We separately tested minority influence on the majority sub-group (5 players) and on the mid-size sub-group (3 players) by running a mixed-effects model with a random effect for condition, nested in the date the game was run. We regressed the treatment dummy (1 if the bot is VAE, 0 if random), round difficulty, and their interaction terms on *Minority Influence*. The results in Table 2 suggests that the VAE bot reduced minority influence on the mid-sized groups in easy rounds (*β* = −0.36, *p* = .03). However, in teams with the VAE bot, minority influence on the majority increased when the majority cluster faced difficult rounds, and thus people were most uncertain (*β* = 0.31, *p* < 0.001).

**Table 2:** Mixed models for minority influence. The VAE bot was successful at increasing minority influence when the majority cluster faced difficult decisions and majority players were thus most uncertain about their responses. The VAE bot was also successful at increasing 2-person minority cluster’s influence on the mid-sized 3-person group.

Dependent Variable:	Minority Influence^majority^		Minority Influence^*mid*−*size*^	
Predictors	Estimate	*p-value*	Estimates	*p-value*
(Intercept)	0.07	0.368	0.22	0.064
Treatment	-0.14	0.181	-0.36*	0.030
Majority Difficulty	0.13*	0.014		
Treatment: Majority Difficulty	0.31***	<0.001		
Mid-Size Difficulty			0.21***	<0.001
Treatment: Mid-Size Difficulty			0.02	0.784
Random effects	Yes		Yes	
Observations	572		572	
Marginal R^2^ / Conditional R^2^	0.101 / 0.128		0.082 / 0.219	

Mirroring the above analysis, we also tested whether the majority was less influential in VAE teams than in control teams (the construction of majority influence variable is described in the Supporting information). We controlled for round difficulty for participants in the non-majority predictor categories (Table 3). We found no main effect of treatment but once again an interaction with round difficulty. In particular, we found a reduced influence of the majority sub-group on the minority sub-group with increasing round difficulty (*β* = −0.14, *p* = .03).

**Table 3:** Mixed models for majority influence. The VAE bot significantly reduced the majority cluster’s influence on the 2-person minority cluster. A significant interaction between treatment and difficult was also found, suggesting that presence of the VAE bot further reduced majority cluster’s influence in difficult trials.

Dependent Variable:	Majority Influence^minority^		Majority Influence^*mid*−*size*^	
Predictors	Estimate	*p-value*	Estimates	*p-value*
(Intercept)	0.07	0.457	0.09	0.457
Treatment	-0.13	0.305	-0.23	0.191
Minority Difficulty	0.21***	<0.001		
Treatment: Minority Difficulty	-0.14*	0.036		
Mid-Size Difficulty			0.13**	0.006
Treatment: Mid-Size Difficulty			0.10	0.112
Random effects	Yes		Yes	
Observations	840		840	
Marginal R^2^ / Conditional R^2^	0.025 / 0.116		0.047 / 0.226	

Overall, these findings show that the minority group had a greater influence on the majority group in VAE teams than in control teams. On the contrary, the majority had less influence on the minority group. These effects were modulated by round difficulty, as expected by the fact that people are more prone to social influence when facing difficult decisions. These findings also suggest that the presence of a VAE bot supporting minority views more than majority views made the minority opinion more resistant to social influence and more influential under challenging rounds.

### Individual prediction accuracy

We tested whether the presence of a minority-supporting bot affected the prediction accuracy of human participants. We ran a regression model to compare changes in prediction errors after social interaction in teams with a VAE algorithmic player versus teams with a randomizing bot. We controlled for other factors affecting prediction error like round difficulty, learning success during the training phase, and quality of social information.

Prediction error was defined as the absolute value of the difference between predictions provided by individual *i* in round *k*, and the round outcome indicator (Rain = 1; No Rain = 0):

$$\begin{array}{c} \text{prediction}\;{\text{error}}_{i,k}=\left|{\text{prediction}}_{i,k}-{\text{outcome}}_{k}\right| \end{array}$$ (9)

Δerror is the change in prediction error from the initial to the final forecast provided by a participant. A larger value of Δerror indicates a larger accuracy improvement. The variable is defined as:

$$\begin{array}{c} {\text{improvement}}_{i,k}=\Delta {\text{error}}_{i,k}=\text{prediction}\;{\text{error}}_{i,k}^{\text{initial}}-\text{prediction}\;{\text{error}}_{i,k}^{\text{final}} \end{array}$$ (10)

where initial and final indicate predictions made before or after seeing other team members’ forecasts.

We identified three explanatory variables that could confound accuracy improvements due to our manipulation, namely (1) the accuracy of other team members’ predictions in a given round (see Supplementary Information §Collective error), (2) the round’s difficulty (see Supplementary Information §Round Difficulty) and (3) how well a participant had learned the relationship between predictor values and rain probability during training (see Supplementary Information §Training Error).

In addition to the above-defined explanatory variables, we also control for the effect of a participant being in either the temperature, humidity, or wind predictor groups. Given that these three predictors have a different underlying relationship with rain probability, we include interaction terms between these indicator group dummy variables and the presence of a VAE bot. Including this interaction term is important since we may expect that our treatment affects participants differently based on which predictor group they belong to. We standardized all continuous variables.

We ran a mixed regression model on Δerror (S6 Table in S1 File). We also report the same model run on the sign of Δerror, Δdummy, representing binary improvement (*i*.*e*. whether accuracy improved or not) and raw final prediction error (representing final round accuracy)—see S7-S9 Tables in S1 File.

The results show a positive trend (*β* = 0.16, *p* = .06) for the VAE bot condition, indicating weak accuracy improvement due to the presence of the VAE bot, and a significant interaction between treatment and wind condition (*β* = −0.26, *p* = 0.03), suggesting that the accuracy improvement was negatively affected by being assigned to a wind predictor (arguably the most difficult predictor-outcome relationship to learn). These results were confirmed when we fitted a logistic mixed model with Δdummy as the outcome variable. Results were robust to an alternative round difficulty measure (absolute value of z-scores).

As expected, training error, collective error, and round difficulty all increased prediction error (*p* < 0.001). Furthermore, a higher collective error decreased the chance that a participant provided an improved forecast after seeing peer forecasts (*p* < 0.001) (S4 Table in S1 File).

To better understand what drove these accuracy improvements, we ran the same models on subsets of our sample based on which predictor group the participant belonged to. The results show that significant improvements due to VAE presence were driven by participants assigned to the humidity condition (S5-S7 Tables in S1 File). As the humidity condition was arguably the easiest to learn (positive linear), we interpret these results as suggesting that greater accuracy improvements were observed in those participants who were more likely to have learned the relationship of their indicator variable with the outcome. When dividing our sample based on which predictor group, we found that VAE significantly increased individual accuracy when the 2-person minority was assigned to the quadratic “wind” indicator variable (S6, S7 Tables in S1 File). In all other models, the only significant predictors of individual accuracy improvement were round difficulty, training error and collective error (S5-S7 Tables in S1 File). Overall, these findings suggest that gains on individual accuracy due to the VAE bot were small but consistent.

A caveat of this analysis is the high attrition rate observed during the initial forecast, which thus reduced the number of observations where a Δerror measure could be computed.

### Team performance

**Fig 4:**
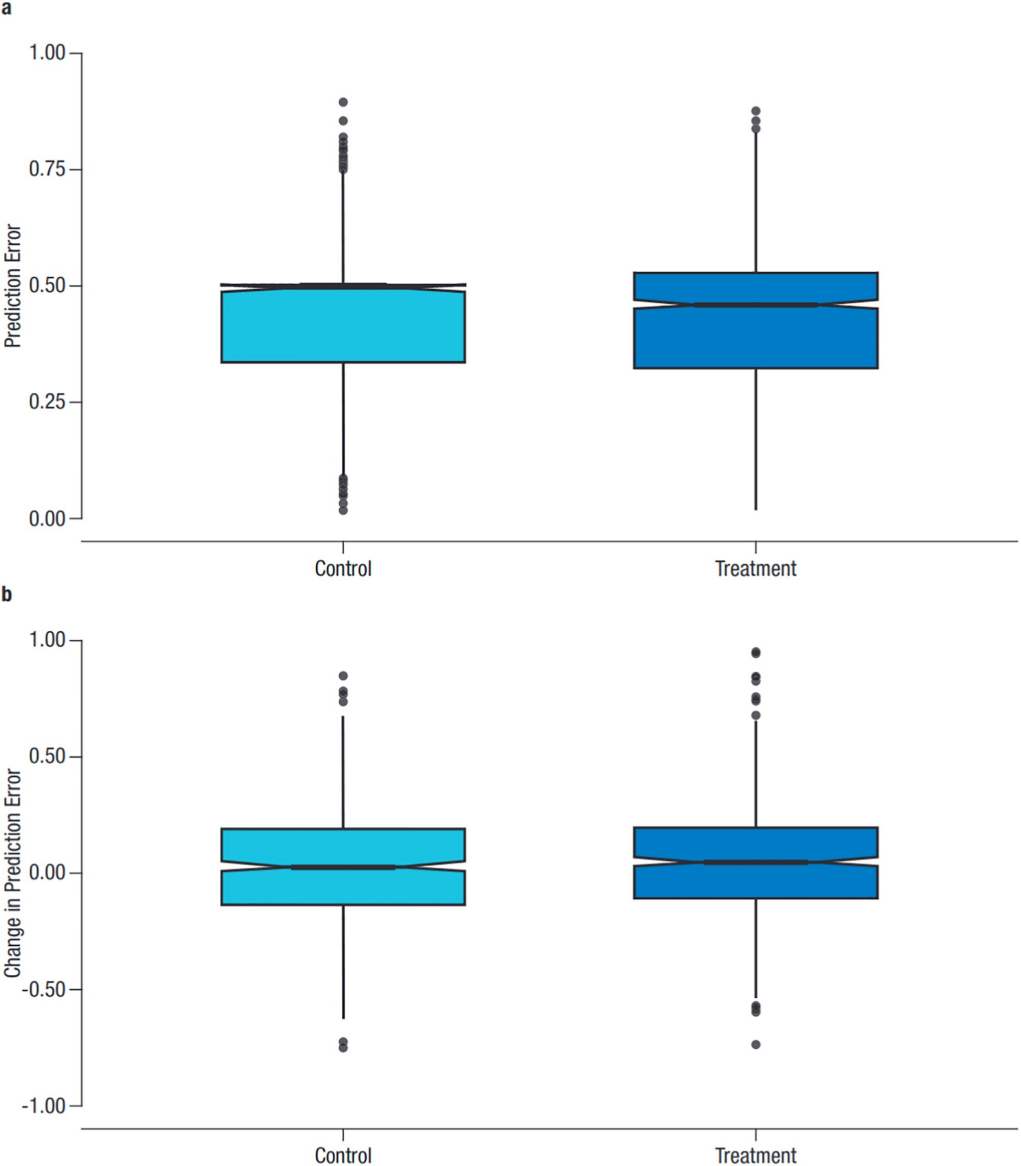
Boxplot of Team Median Prediction Errors (top panel) and the Difference between Initial and Final Median Prediction Error (bottom panel).

Further to the above models, where we look at round-level observations from individual participants, we tested for differences in team accuracy between control and treatment teams. We defined team accuracy as the median team prediction error. When looking at aggregate data, we did not observe any significant difference in median error between treatment teams and median increase in prediction accuracy compared to control teams (both using a two-sample t-test (*p* >.05) and a Wilcoxon test (*p* >.05) (Fig 4).

We used two dependent variables when testing whether our treatment significantly affected team performance: median team error and change in median team error. We calculated the former in a similar way as we calculated prediction error for round-level individual forecasts. We took the median prediction from a team and calculated the error from this prediction. For the analysis, we are only interested in the effect of bot treatment on team accuracy exclusive of bot accuracy. For comparison, however, we report results both when predictions by bots were excluded (*median error*) and included (*median error (bot)*).

$$\begin{array}{c} \text{median}\;{\text{error}}_{j,k}=\left|\text{med}\left({\text{prediction}}_{j,k}^{\text{final}}\right)-{\text{outcome}}_{k}\right| \end{array}$$ (11)

Our second variable of interest was Δ *median error*, which measured the improvement from the median initial prediction to the median final prediction. The variable was computed for team *j* in round *k* as follows:

$$\begin{array}{c} \Delta \text{median}\;{\text{error}}_{j,k}=\left|\text{med}\left({\text{prediction}}_{j,k}^{\text{initial}}\right)-{\text{outcome}}_{k}\right|-\left|\text{med}\left({\text{prediction}}_{j,k}^{\text{final}}\right)-{\text{outcome}}_{k}\right| \end{array}$$ (12)

Similarly to median error, bot predictions were excluded for Δ*median error* and were included for Δ*median error (bot)*.

We controlled for the same variables that were used in individual-level accuracy. Initial collective error was measured by the same variable as in the individual mixed-effects models *Collective Error*, while round difficulty was simply the weighted average of the *Round Difficulty* measure used at the individual level. The weights of wind, temperature and humidity difficulty were based on the number of people in each sub-group. The sample calculation method for a team with five participants in the wind, three in humidity and two in the temperature condition was (5 × Round Difficulty Wind + 3 × Round Difficulty Hum + 2 × Round Difficulty Temp)/10.

To control for training success, we summed up all the prediction errors of the last 55 training rounds in a team, and then divided the sum by 550 (55 rounds × 10 participants per team):

$$\begin{array}{c} \text{training}\;{\text{error}}_{j}=\frac{\sum_{k=56}^{110}\sum_{i=1}^{10}\text{prediction}\;{\text{error}}_{i,k}}{550} \end{array}$$ (13)

Where *i* ∈ *I*_*j*_ denotes participants in team *j*.

In addition to the above three control variables, we included interaction terms between the treatment variable and two further dummy variables indicating whether a group had two participants (the smallest possible group) in the wind condition or in the temperature condition. The models included a random effect for team, nested in the date the game took place. The full model is reported in S8 Table in S1 File.

The results presented in Table 4 show a marginal negative effect of our treatment on team *medianerror* (*p* <.1) for teams when temperature was assigned to the smallest minority group (N = 2). However, this effect was not replicated with Δ*median error*.

**Table 4:** Mixed model estimates of treatment effect on team forecasts.

Dependent Variable:	*median error*		*median error (bot)*		Δ *median error*		Δ *median error (bot)*	
Predictors	Estimate	*p-value*	Estimate	*p-value*	Estimate	*p-value*	Estimate	*p-value*
(Intercept)	-0.10	0.380	-0.10	0.332	0.12	0.212	0.09	0.319
Treatment	0.24	0.153	0.19	0.249	-0.17	0.266	-0.17	0.205
Temp. Minority	0.18	0.311	0.17	0.326	-0.26’	0.096	-0.13	0.348
Treatment: Temp. Minority	-0.49’	0.083	-0.38	0.173	0.38	0.133	0.28	0.207
Wind Minority	0.13	0.374	0.15	0.292	-0.25’	0.059	-0.12	0.287
Treatment: Wind Minority	-0.29	0.163	-0.24	0.242	0.35’	0.067	0.22	0.194
Random effects	Yes		Yes		Yes		Yes	
Observations	1500		1500		1290		1500	
Marginal / Conditional R^2^	0.109 / 0.150		0.150 / 0.189		0.165 / 0.193		0.470 / 0.495	

These findings suggest that wind minority groups in the random control condition got worse after social exposure. A trend was observed indicating that our treatment marginally reversed this effect, thus positively affecting accuracy improvements in this condition (*p* = .06). Overall, these findings suggest that improvements on team accuracy were weak and constrained by what indicator variable the 2-person minority group was assigned to. However, the VAE bot had a stronger effect in conditions when the 2-person minority cluster was assigned to the quadratic indicator (“wind”).

## 4 Discussion

In this paper, we designed a strategic algorithmic player and deployed it along with human players in hybrid teams performing a hidden-profile task. The bot was built on a variational-autoencoder trained to learn a low-dimensional representation of opinion clusters based on the repeated observations of people’s judgments. The bot was programmed to generate original predictions on the fly that supported minority views more than majority views. We manipulated the size and information of three subgroups, namely a majority (5-person condition) and two minority subgroups (3 and 2-person conditions, respectively), with access to unique task-relevant information.

We expected a bot so designed could bridge the gap between opinion clusters and improve team accuracy. Instead, we found that the presence of a single minority supporting bot (representing only 10% of the team size) increased the polarization of the team compared to a baseline condition where a control algorithmic player provided random guesses. Our original hypothesis was based on studies in social psychology, showing that people are most influenced by opinions that are in a “latitude of acceptance”, *i*.*e*. a Goldilocks region that is not too close to their privately held opinion (not inducing any opinion shift) and not too distant from it (too distant to integrate with one’s information) [4, 36]. We find evidence for an increase in the polarization of the team after the introduction of the VAE bot. This result suggests that rather than bridging the gap and facilitating consensus reaching, the VAE player likely had the unintended consequence of making the minority more resilient to social influence from the majority. This result echoes some early results on persuasion and peer pressure showing that the likelihood of yielding to a majority believed to be wrong dramatically decreases with each additional minority-supporting individual [1, 37]. Exposure to disagreeing opinions is rarely successful at changing one’s beliefs or confidence and can sometimes have the opposite effect of entrenching people in their views event further [19, 38–41]. For this reason, bots may be more effective at increasing polarization than at changing opinions [42, 43].

On the other hand, the VAE bot was unsuccessful in increasing the influence of participants in the minority conditions on the majority, suggesting that participants simply became more entrenched in their views. However, we also find weak but consistent evidence that the bot affected collective decision patterns in subtle ways. We found weak evidence that the VAE bot helped participants improve accuracy from their initial to final forecasts. S6 Table in S1 File shows that observations from temperature minority groups drove this effect. These results suggest that all else being equal, minority participants assigned the temperature indicator (which had a negative linear relationship with the outcome) improved their accuracy thanks to the VAE presence more than their counterparts in the control. Our findings also show weak evidence that the VAE bot influenced team performance, although these effects did not reach significance. The VAE bot numerically reduced final forecasting errors and error change from initial to final forecast for specific teams (for instance, in teams when the minority was assigned to a negatively linear ‘temperature’ indicator). However, the weak effects make the interpretation of accuracy results difficult. Considering that our bot treatment represented only 10% of the entire team, these results are not surprising. Future experiments should include a larger number of strategic bots and/or stronger biases towards minority opinions [1, 37].

More importantly, our VAE algorithmic agent inferred group membership without being provided any information other than the number of clusters (*k*) to partition human players. However, contrary to simple clustering algorithms, using a variational auto-encoder made it possible to sample new unseen responses from the hidden layer representing other players. This is in stark contrast with bots that simply copy human behavior. Variational auto-encoders reduce redundancy in the input similarly to principal component analysis. Thus, the VAE model in our experiment likely inferred group membership by leveraging on the correlation of people forecasts over time. The model then used this learned internal representation to place itself along this hidden space to support minority views proportionally more than majority views. This strategy was intended to maximally influence collective dynamics by facilitating consensus reaching. Although our algorithmic manipulation was not successful at reducing conflict, it did influence team dynamics, from increasing polarization to influencing individual and collective patterns of decision accuracy.

The growing accessibility of machine learning tools and large amounts of data has significantly increased the sophistication of algorithmic agents online. Arguably, online platforms are more likely to block unsophisticated bots than bots that are better able to mimic human behavior and opinions. This trend might, over time, create a selection process favoring ‘more human’ sophisticated bots. Here, we explored how adaptive algorithmic agents (using off-the-shelf machine learning techniques) could affect team dynamics and decision-making in a controlled environment. The consequences of smarter bots on collective dynamics are largely unknown. Understanding them will require conducting more experiments like the present work. Randomized treatments can shed light on these complex dynamics in the lab and larger field experiments on real online platforms. Even though our bot was explicitly designed to improve team performance in the hidden-profile task, the results were surprising. Our findings suggest that algorithms in hybrid teams interact in complex ways with how people perceive, share and integrate information to solve common goals.

These findings have profound implications for social science. In today’s information environment, people’s beliefs and political orientation predict media consumption, health-related behaviors, and social networks [44–46]. Correlation, in turn, can reduce groups’ accuracy [27–29]. Our findings highlight that shared information sources can produce correlation patterns in people’s beliefs that algorithmic agents can exploit to infer group membership and increase polarization between groups holding different beliefs.

However, our findings also show that algorithms can positively impact the collective information landscape. Algorithmic agents can be designed to de-correlate a group’s information pattern by artificially supporting minority views. In the case of minority groups who hold rare but useful information (like in the case of the hidden profile paradigm), algorithms can benefit group dynamics. Minority views are often difficult to be integrated into group decision-making, and voting mechanisms can be biased to support majority groups [47]. Various mechanisms have been suggested to alleviate this problem, including deliberation, modular aggregation, and quadratic voting [48, 49]. Our findings show how algorithms could help support independent but minority views, potentially leading to fairer outcomes for less powerful groups.

Notwithstanding the novelty of the results, we must stress that our analysis was exploratory, and many caveats exist in our paradigm. First and foremost, we observed a large rate of non-compliance when participants were asked to provide initial forecasts. This phenomenon may have been driven by decision fatigue or by the fact that participants in our study were not rewarded for providing accurate initial forecasts but only accurate final forecasts. Providing a performance bonus to the top two ranked participants in a team may have reduced the incentive for team members to contribute to a shared pool of knowledge, given that this would only make other participants more likely to achieve a higher score. Decision fatigue may also be a reason why we saw few initial guesses. The experimental setting required less effort to look at what other participants in the team guessed before providing any forecast compared to forecasting from the initial private information. In short, the attentional and cognitive cost of providing initial forecasts seem to have been greater than any associated benefit.

Finally, given that the bots provided initial forecasts in each round, the lack of initial guesses from human participants may have artificially increased the influence of bot forecasts on our findings. This may result in an inflated effect size compared to a scenario where all participants provide initial forecasts. Although far from perfect, we wish our methodology could stimulate more experimental work to understand these phenomena. As seen in this work, subtle and unexpected effects may come from using off-the-shelf machine learning algorithms in hybrid social systems.

## 5 Conclusions

The increased sophistication of social bots and the availability of off-the-shelf machine learning algorithms are likely to create a generation of smart algorithmic agents able to map opinion clusters and actively infer group membership. Generative models such as variational auto-encoders and generative adversarial networks can be used to produce bots that can strategically position themselves along arbitrary opinion spaces to maximally influence collective dynamics. In this paper, we explored how a single algorithmic agent—implemented using readily available machine learning tools– can be used to solve the hidden-profile task by strategically supporting minority views against a dominant majority.

## Supporting information

S1 Fig Predictive functions. Predictive functions on the [0, 1] interval (pink horizontal line represents the function average on this interval). Notice that the quadratic function was limited to 2 and -2. The reason behind this decision was empirical. Pilot data showed that a smooth quadratic relation was difficult to learn. We thus decided to make it more extreme, so to make it clearer that high and low values of wind predicted rain, while values that were close to 50% were predictive of no-rain.
(PNG)

S2 Fig Sigmoid function calculating rain probability. Sigmoid Function Calculating Rain Probability from normalized predictive functions.
(PNG)

S3 Fig Prediction error by algorithmic players. Prediction Error by Algorithmic players: Random bots vs. VAE (separated by which predictor group was in the minority).
(PNG)

S4 Fig Histogram of predictions. Histogram of Predictions: Random *vs*. VAE.
(PNG)

S1 File Supplementary tables. (PDF)

## References

[1] Asch Solomon E. Studies of Independence and Conformity: a Minority of One Against a Unanimous Majority. *Psychological Monographs: General and Applied*, 70(9, whole no. 416):1–70, 1956. doi: 10.1037/h0093718

[2] Flache Andreas, Mäs Michael, Feliciani Thomas, Chattoe-Brown Edmund, Deffuant Guillaume, Huet Sylvie, et al. Models of Social Influence: Towards the Next Frontiers. *Journal of Artificial Societies and Social Simulation*, 20(4), 2017. doi: 10.18564/jasss.3521

[3] Kelman HC. Compliance, identification, and internalization: Three processes of attitude change. *Journal of Conflict Resolution*, 2(1):51–60, 1958. doi: 10.1177/002200275800200106

[4] Sherif C.W., Sherif M.S., and Nebergall R.E. *Attitude and attitude change*. W.B. Saunders Company, Philadelphia, 1965.

[5] Alessandro Bessi and Emilio Ferrara. Social Bots Distort the 2016 US Presidential Election Online Discussion. *SSRN*, 21(11), 2016.

[6] Kakutani Michiko. *The Death of Truth: Notes on Falsehood in the Age of Trump*. Harper Collins, 2018.

[7] Onur Varol, Emilio Ferrara, Clayton Davis, Filippo Menczer, and Alessandro Flammini. Online Human-Bot Interactions: Detection, Estimation, and Characterization Authors. In *Eleventh International AAAI Conference on Web and Social Media*, page Vol. 11 No. 1, 2017.

[8] Subrahmanian V.S., Azaria Amos, Durst Skylar, Kagan Vadim, Galstyan Aram, Lerman Kristina, et al. The DARPA Twitter Bot Challenge. *Computer*, 49(6):38–46, 6 2016. doi: 10.1109/MC.2016.183

[9] Stefan Stieglitz, Florian Brachten, Björn Ross, and Anna-Katharina Jung. Do Social Bots Dream of Electric Sheep? A Categorisation of Social Media Bot Accounts. 10 2017.

[10] Dunham Ken and Melnick Jim. *Malicious Bots: An Inside Look into the Cyber-Criminal Underground of the Internet*. CRC Press, Boca Raton, 2009.

[11] Lerman Kristina, Yan Xiaoran, and Wu Xin-Zeng. The “Majority Illusion” in Social Networks. *PLOS ONE*, 11(2):e0147617, 2 2016. doi: 10.1371/journal.pone.0147617 26886112 PMC4757419

[12] Malte F. Jung, Nikolas Martelaro, and Pamela J. Hinds. Using Robots to Moderate Team Conflict. In *Proceedings of the Tenth Annual ACM/IEEE International Conference on Human-Robot Interaction*, New York, NY, USA, 3 2015. ACM.

[13] Brinkmann Levin, Gezerli Deniz, von Kleist Kira, Thomas F. Müller, Iyad Rahwan, and Pescetellim Niccolò. Human biases limit algorithmic boosts of cultural evolution. *Philosophical Transactions of the Royal Society A*, 2021.

[14] Stasser Garold and Titus William. Hidden Profiles: A Brief History. *Psychological Inquiry*, 14(3):304–313, 2003. doi: 10.1207/S15327965PLI1403&4_21

[15] Lightle John P., Kagel John H., and Arkes Hal R. Information Exchange in Group Decision Making: The Hidden Profile Problem Reconsidered. *Management Science*, 55(4):568–581, 4 2009. doi: 10.1287/mnsc.1080.0975

[16] Stella Massimo, Ferrara Emilio, and De Domenico Manlio. Bots increase exposure to negative and inflammatory content in online social systems. *Proceedings of the National Academy of Sciences of the United States of America*, 115(49):12435–12440, 12 2018. doi: 10.1073/pnas.1803470115 30459270 PMC6298098

[17] Jacob Ratkiewicz, Michael Conover, Mark Meiss, Bruno Goncalves, Alessandro Flammini, and Filippo Menczer. Detecting and Tracking Political Abuse in Social Media. In *Proceedings of the International AAAI Conference on Web and Social Media*, page 5(1), 2011.

[18] Stefano Cresci, Roberto Di Pietro, Marinella Petrocchi, Angelo Spognardi, and Maurizio Tesconi. The Paradigm-Shift of Social Spambots. In *Proceedings of the 26th International Conference on World Wide Web Companion—WWW’17 Companion*, pages 963–972, New York, New York, USA, 2017. ACM Press.

[19] Bail Christopher A., Argyle Lisa P., Brown Taylor W., Bumpus John P., Chen Haohan, Fallin Hunzaker M. B., et al. Exposure to opposing views on social media can increase political polarization. *Proceedings of the National Academy of Sciences*, 115(37):9216–9221, 9 2018. doi: 10.1073/pnas.1804840115 30154168 PMC6140520

[20] Bail Christopher A., Guay Brian, Maloney Emily, Combs Aidan, Sunshine Hillygus D., Merhout Friedolin, et al. Assessing the Russian Internet Research Agency’s impact on the political attitudes and behaviors of American Twitter users in late 2017. *Proceedings of the National Academy of Sciences*, 117(1):243–250, 1 2020. doi: 10.1073/pnas.1906420116 31767743 PMC6955293

[21] Allen Jennifer, Howland Baird, Mobius Markus, Rothschild David, and Watts Duncan J. Evaluating the fake news problem at the scale of the information ecosystem. *Science Advances*, 6(14):eaay3539, 4 2020. doi: 10.1126/sciadv.aay3539 32284969 PMC7124954

[22] Guess Andrew, Nagler Jonathan, and Tucker Joshua. Less than you think: Prevalence and predictors of fake news dissemination on Facebook. *Science Advances*, 5(1):eaau4586, 1 2019. doi: 10.1126/sciadv.aau4586 30662946 PMC6326755

[23] Philip Howard. How Political Campaigns Weaponize Social Media Bots. *IEEE Spectrum*, Oct, 2018.

[24] Ferrara Emilio, Varol Onur, Davis Clayton, Menczer Filippo, and Flammini Alessandro. The rise of social bots. *Communications of the ACM*, 59(7):96–104, 6 2016. doi: 10.1145/2818717

[25] Ledford Heidi. Social scientists battle bots to glean insights from online chatter. *Nature*, 578(7793):17–17, 2 2020. doi: 10.1038/d41586-020-00141-1 32020108

[26] Hahn Ulrike, von Sydow Momme, and Merdes Christoph. How Communication Can Make Voters Choose Less Well. *Topics in Cognitive Science*, 11(1):194–206, 1 2019. doi: 10.1111/tops.12401 30585433

[27] Ladha Krishna K. The Condorcet Jury Theorem, Free Speech, and Correlated Votes. *American Journal of Political Science*, 36(3):617, 8 1992. doi: 10.2307/2111584

[28] Kao Albert B., Miller Noam, Torney Colin, Hartnett Andrew, and Couzin Iain D. Collective Learning and Optimal Consensus Decisions in Social Animal Groups. *PLoS Computational Biology*, 10(8):e1003762, 8 2014. doi: 10.1371/journal.pcbi.1003762 25101642 PMC4125046

[29] Lorenz Jan, Rauhut Heiko, Schweitzer Frank, and Helbing Dirk. How social influence can undermine the wisdom of crowd effect. *Proceedings of the National Academy of Sciences of the United States of America*, 108(22):9020–5, 5 2011. doi: 10.1073/pnas.1008636108 21576485 PMC3107299

[30] Higgins Irina, Chang Le, Langston Victoria, Hassabis Demis, Summerfield Christopher, Tsao Doris, et al. Unsupervised deep learning identifies semantic disentanglement in single inferotemporal face patch neurons. *Nature Communications*, 12(1):6456, 12 2021. doi: 10.1038/s41467-021-26751-5 34753913 PMC8578601

[31] Orabi Mariam, Mouheb Djedjiga, Aghbari Zaher Al, and Kamel Ibrahim. Detection of Bots in Social Media: A Systematic Review. *Information Processing & Management*, 57(4):102250, 7 2020. doi: 10.1016/j.ipm.2020.102250

[32] Shirado Hirokazu and Christakis Nicholas A. Locally noisy autonomous agents improve global human coordination in network experiments. *Nature*, 545(7654):370–374, 5 2017. doi: 10.1038/nature22332 28516927 PMC5912653

[33] Almaatouq Abdullah, Becker Joshua, Houghton James P., Paton Nicolas, Watts Duncan J., and Whiting Mark E. Empirica: a virtual lab for high-throughput macro-level experiments. *Behavior Research Methods*, 53(5):2158–2171, 10 2021. doi: 10.3758/s13428-020-01535-9 33782900 PMC8516782

[34] Brier Genn W. Verification of Forecasts Expressed in Terms of Probability. *Monthly Weather Review*, 78(1):1–3, 1 1950. doi: 10.1175/1520-0493(1950)078<0001:VOFEIT>2.0.CO;2

[35] Bramson Aaron, Grim Patrick, Singer Daniel J., Fisher Steven, Berger William, Sack Graham, et al. Disambiguation of social polarization concepts and measures. *The Journal of Mathematical Sociology*, 40(2):80–111, 4 2016. doi: 10.1080/0022250X.2016.1147443

[36] Yaniv Ilan and Milyavsky Maxim. Using advice from multiple sources to revise and improve judgments. *Organizational Behavior and Human Decision …*, 103:104–120, 2007. doi: 10.1016/j.obhdp.2006.05.006

[37] Latane Bibb. The psychology of social impact. *American Psychologist*, 36(4):343–356, 1981. doi: 10.1037/0003-066X.36.4.343

[38] Pescetelli Niccolò and Yeung Nick. The effects of recursive communication dynamics on belief updating. *Proceedings of the Royal Society B: Biological Sciences*, 287(1931):20200025, 7 2020. doi: 10.1098/rspb.2020.0025 32693730 PMC7423656

[39] Zaller John. *The Nature and Origins of Mass Opinion*. Cambridge University Press, Cambridge, UK, 1992.

[40] Endres Kyle and Panagopoulos Costas. Cross-Pressure and Voting Behavior: Evidence from Randomized Experiments. *The Journal of Politics*, 81(3):1090–1095, 7 2019. doi: 10.1086/703210

[41] Kalla Joshua L. and Broockman David E. The Minimal Persuasive Effects of Campaign Contact in General Elections: Evidence from 49 Field Experiments. *American Political Science Review*, 112(1):148–166, 2 2018. doi: 10.1017/S0003055417000363

[42] Leo G. Stewart, Ahmer Arif, and Kate Starbird. Examining Trolls and Polarization with a Retweet Network. In *Proc. ACM WSDM*, *Workshop on Misinformation and Misbehavior Mining on the Web*, 2018.

[43] Broniatowski David A., Jamison Amelia M., Qi SiHua, AlKulaib Lulwah, Chen Tao, Benton Adrian, et al. Weaponized Health Communication: Twitter Bots and Russian Trolls Amplify the Vaccine Debate. *American Journal of Public Health*, 108(10):1378–1384, 10 2018. doi: 10.2105/AJPH.2018.304567 30138075 PMC6137759

[44] de Bruin Wändi Bruine, Saw Htay-Wah, and Goldman Dana P. Political polarization in US residents’ COVID-19 risk perceptions, policy preferences, and protective behaviors. *Journal of Risk and Uncertainty*, 61(2):177–194, 10 2020. doi: 10.1007/s11166-020-09336-3 33223612 PMC7672261

[45] Mark Jurkowitz, Amy Mitchell, Elisa Shearer, and Mason Walker. U.S. Media Polarization and the 2020 Election: A Nation Divided. Technical report, 2020.

[46] Guilbeault Douglas, Becker Joshua, and Centola Damon. Social learning and partisan bias in the interpretation of climate trends. *Proceedings of the National Academy of Sciences*, 115(39):9714–9719, 9 2018. doi: 10.1073/pnas.1722664115 30181271 PMC6166837

[47] Moeckli Daniel. Referendums: Tyranny of the Majority? *Swiss Political Science Review*, 24(3):335–341, 9 2018. doi: 10.1111/spsr.12317

[48] Kao Albert B. and Couzin Iain D. Modular structure within groups causes information loss but can improve decision accuracy. *Philosophical Transactions of the Royal Society B: Biological Sciences*, 374(1774):20180378, 6 2019. doi: 10.1098/rstb.2018.0378 31006371 PMC6553586

[49] Eric Posner and Glenn Weil. *Radical Markets: Uprooting Capitalism and Democracy for a Just Society*. 2018.

