# Supplementary figures and images for: A variational-autoencoder approach to solve the hidden profile task in hybrid human-machine teams
Source: PLoS One. 2022 Aug 2;17(8):e0272168. doi: 10.1371/journal.pone.0272168 (PMC9345362; doi:10.1371/journal.pone.0272168)

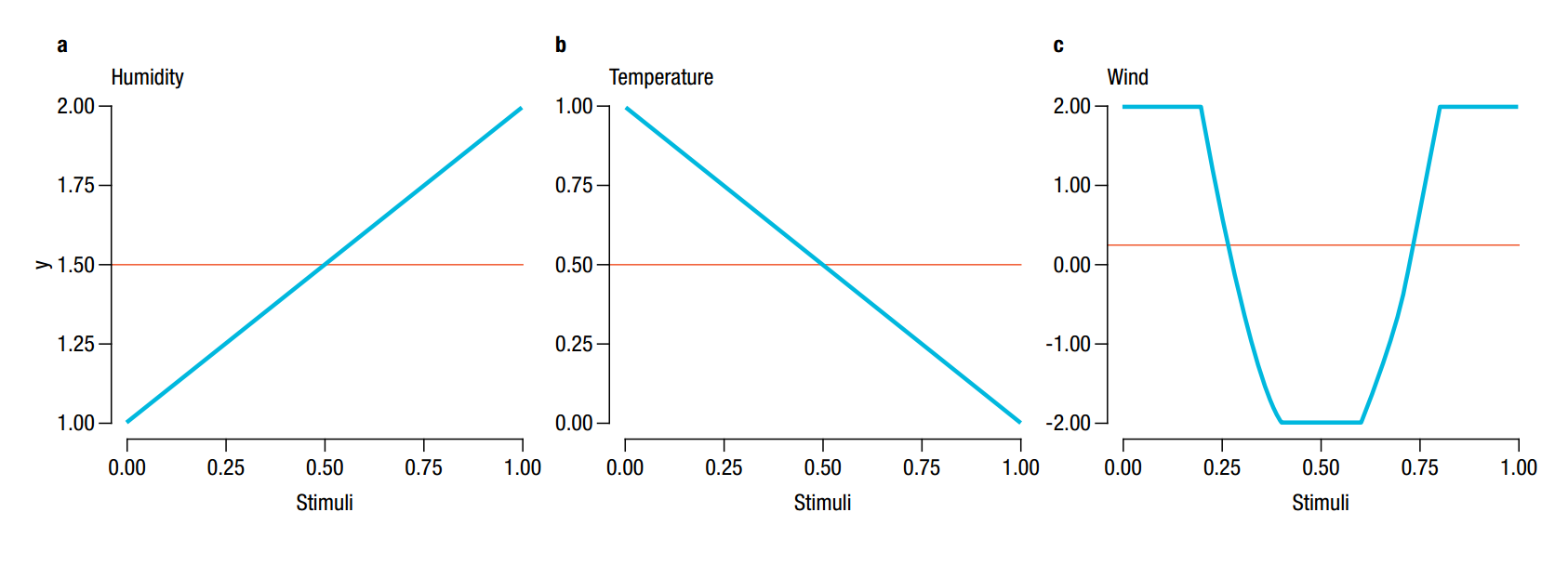

Supplement: S1 Fig — Predictive functions on the [0, 1] interval (pink horizontal line represents the function average on this interval). Notice that the quadratic function was limited to 2 and -2. The reason behind this decision was empirical. Pilot data showed that a smooth quadratic relation was difficult to learn. We thus decided to make it more extreme, so to make it clearer that high and low values of wind predicted rain, while values that were close to 50% were predictive of no-rain. (PNG) [file pone.0272168.s001.png]

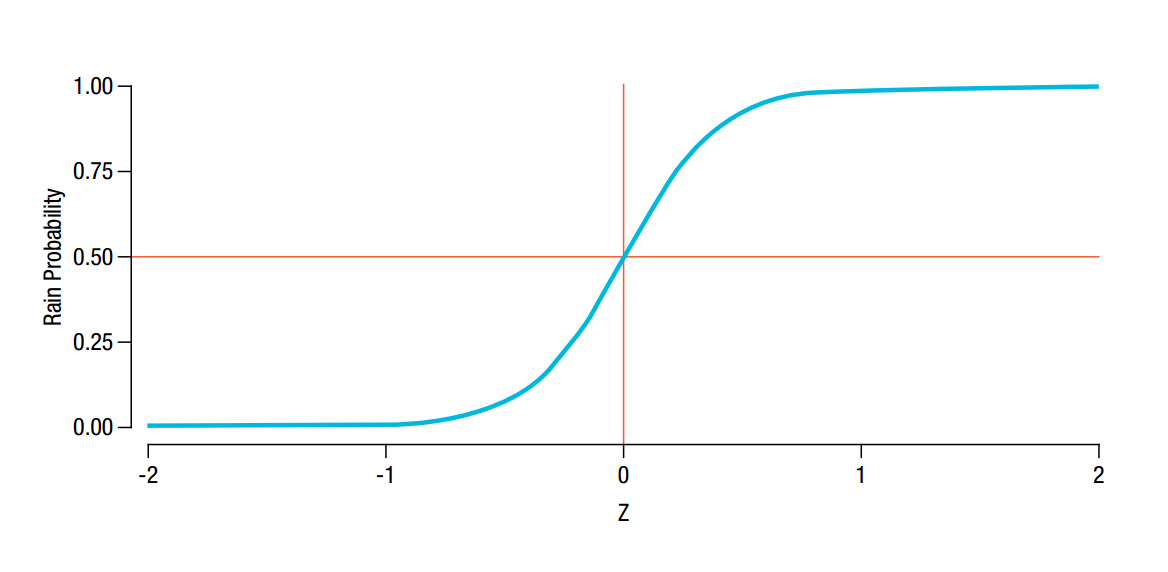

Supplement: S2 Fig — Sigmoid Function Calculating Rain Probability from normalized predictive functions. (PNG) [file pone.0272168.s002.png]

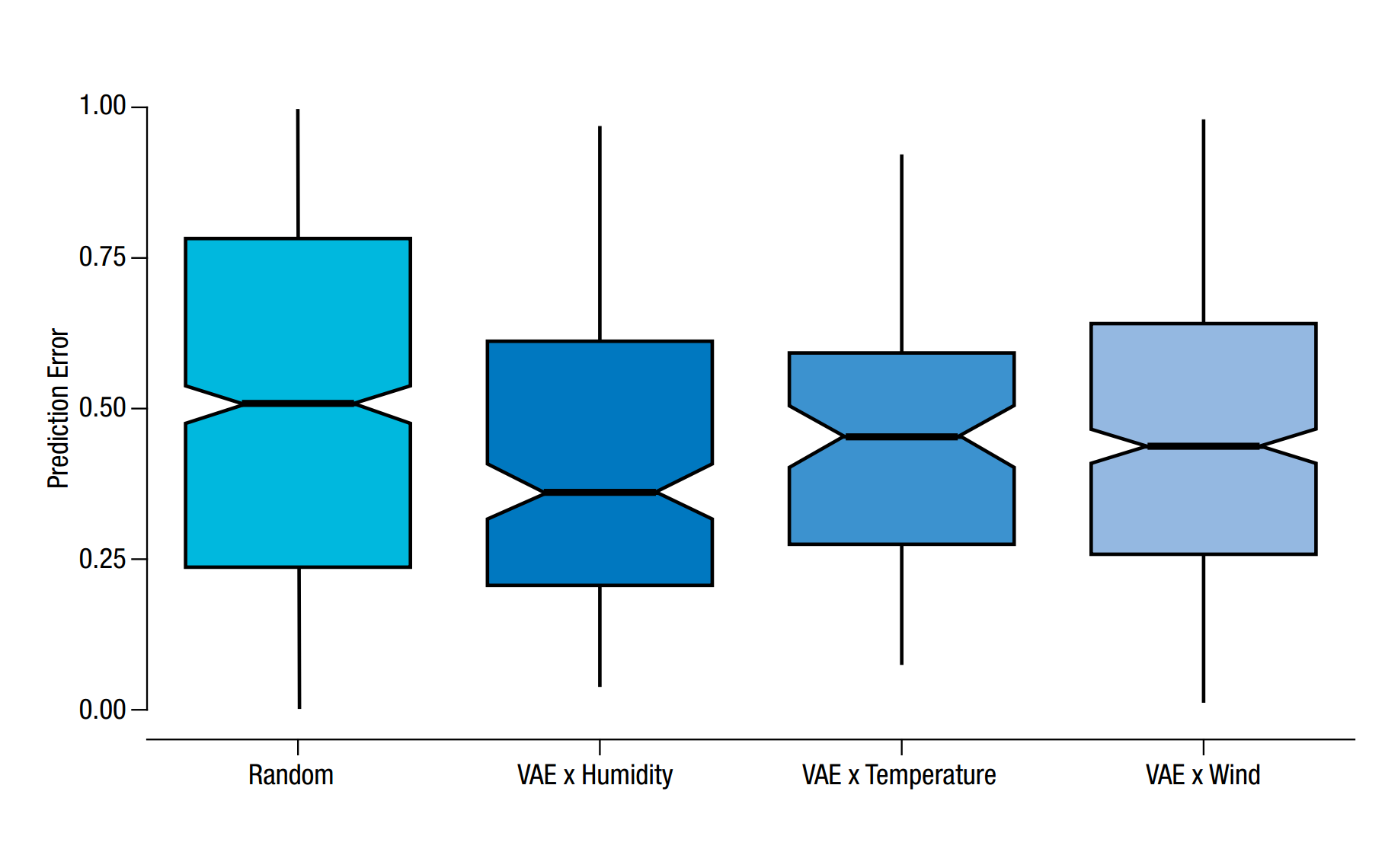

Supplement: S3 Fig — Prediction Error by Algorithmic players: Random bots vs. VAE (separated by which predictor group was in the minority). (PNG) [file pone.0272168.s003.png]

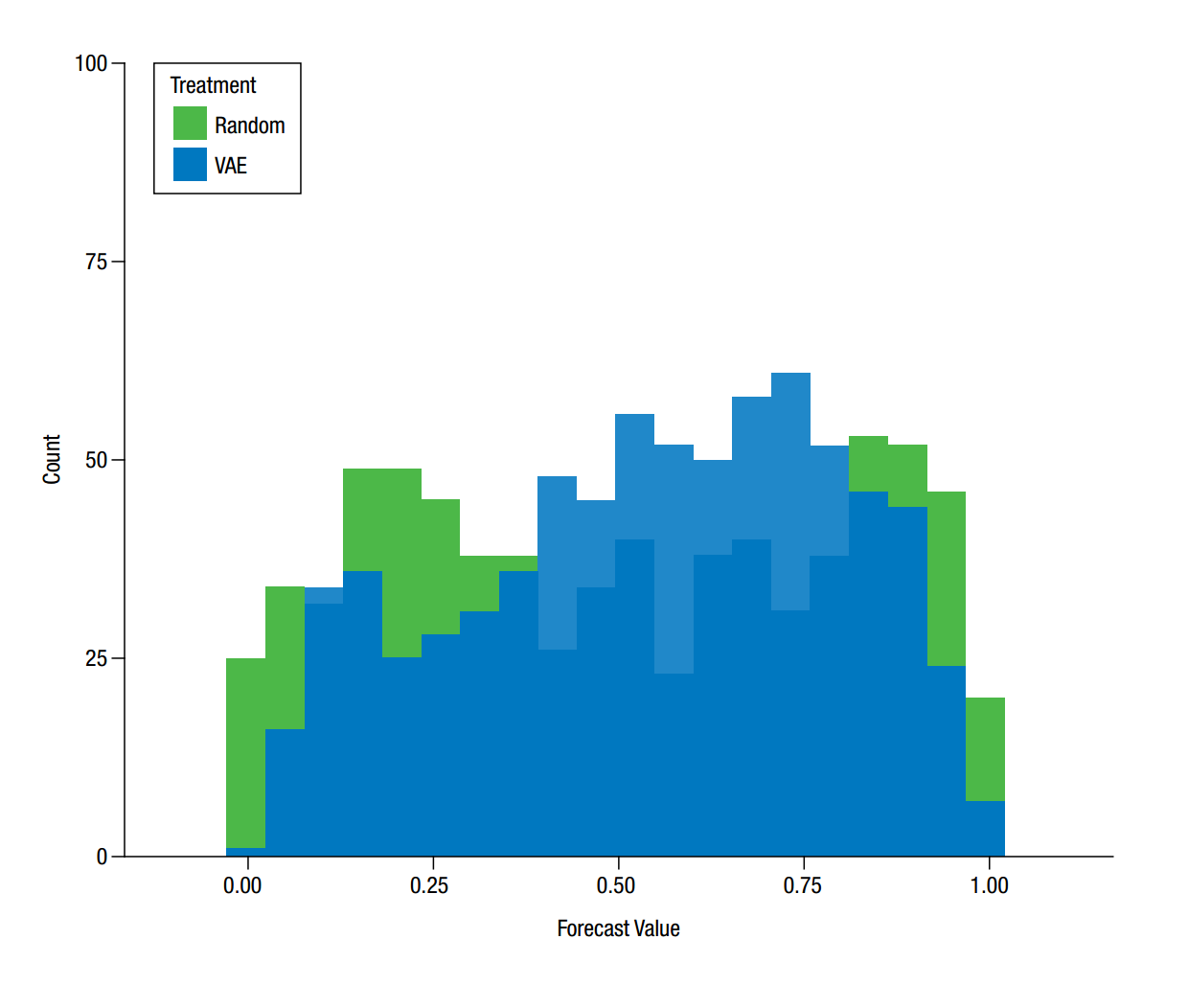

Supplement: S4 Fig — Histogram of Predictions: Random vs. VAE. (PNG) [file pone.0272168.s004.png]
