# Supplementary material for: A variational-autoencoder approach to solve the hidden profile task in hybrid human-machine teams
Source: PLoS One. 2022 Aug 2;17(8):e0272168. doi: 10.1371/journal.pone.0272168 (PMC9345362; doi:10.1371/journal.pone.0272168)
Supplement: S1 File — (PDF) [file pone.0272168.s005.pdf]

4 Supporting Information

Brier score

Participants were incentivised to truthfully report their beliefs by using an incentive mechanism based on the Brier score. We computed Brier scores using the equation proposed in the original paper by Glenn Brier:

$$b = (I - p)^2 + ((1 - I) - (1 - p))^2 \tag{S1}$$

Where I is the indicator variable (I=0: sun; I=1: rain), and p is the probabilistic forecast. The terms (1-I) and (1-p) represent not observing the event and the probability assigned to not observing the event, respectively. The score, ranging from 0 to 2, was used to incentivise truthful reporting in participants but was not used in the subsequent data analysis.

VAE Bot versus Random Forecasts

As a start of our analysis, we want to understand how the VAE model performs versus randomly generated guesses. Figure **??** shows that VAE bots provide lower prediction error than randomized answers, with the 95% confidence intervals of the median not overlapping for bots trained in humidity and wind groups. The boxplots show that VAE with humidity predictor group as the minority performs the best, while wind and temperature perform approximately the same.

To test formally if the trained model has better accuracy compared to random predictions, we run a simple OLS estimation where we regress the treatment dummy ("VAE", 1 if prediction was provided by the VAE algorithm, 0 if it was provided by the randomizing bot), average round difficulty, and dummies for wind and humidity minority on prediction error. The significant ($p < 0.001$) negative coefficient on the treatment dummy indicates that the VAE model provides forecasts with lower prediction error (see Table S2 for results), representing an improvement of 11.5%.

We can observe that the trained model's predictions are closer to a bell-shaped distribution with a positive skew, while the randomized predictions are more skewed towards the extremes (see Figure **??**).

Round Difficulty

The predictor functions include a random noise term, which we ignore for the below calculations. As the z-score of predictor values determines if it has a negative (negative z-score) or positive (positive z-score) effect on rain probability, we just need to find the predictor value that corresponds to the predictor function's average on the $[0, 1]$ interval. Humidity and temperature have a linear positive, and linear negative relationship with rain probability, respectively. The linear relationship makes it straight-forward to calculate which predictor value provides the least amount of information to participants. The average of an either decreasing or increasing linear function on the 0,1 interval is at $x = 0.5$, which is our most ambiguous stimuli value for both humidity and temperature predictors. As wind has a quadratic relationship with rain probability, we will have not one, but two "most ambiguous" values, denoted by x_1 and x_2.

The wind predictor function is given by $f(x) = 50x2 - 50x + 10 + \epsilon$, where $x \in [0, 1]$ and $\epsilon \sim N(0, 1)$, but $f(x) = 2$ if $x \leq 2$ and $0.8 \leq x \leq 1$, furthermore, $f(x) = -2$ if $x \leq -2$. The alternative representation of this function (setting aside the random noise term) is:

$$f(x) = \begin{cases} 2, & 0 \leq x < 0.2 \\ 50x^2 - 50x + 10, & 0.2 \leq x < 0.4 \\ -2, & 0.4 \leq x < 0.6 \\ 50x^2 - 50x + 10, & 0.6 \leq x < 0.8 \\ 2, & 0.8 \leq x \leq 1 \end{cases} \tag{S2}$$

When calculating the function average for $f(x)$ on interval [a,b], we use the following well-known formula:

$$\bar{f}_{a,b} = \frac{1}{b - a} \int_a^b f(x)dx \tag{S3}$$

Following the above calculations, we obtain $x_1 = 0.264755$ and $x_2 = 0.735245$. After we calculated the most ambiguous stimuli for all three predictor categories, we then measure round difficulty by the relative distance of the

stimuli from the most ambiguous stimulus or stimuli (note that the calculation of wind difficulty depends on which interval the wind stimuli belongs to):

$$\text{Round Difficulty}_{i,k} = \begin{cases} 1 - |0.5 - \text{hum}_k|/0.5, & \text{predictor group}_i = \text{humidity} \\ 1 - |0.5 - \text{temp}_k|/0.5, & \text{predictor group}_i = \text{temperature} \\ \text{Wind Difficulty}_k, & \text{predictor group}_i = \text{wind} \end{cases} \tag{S4}$$

Where Wind Difficulty is calculated as:

$$\text{Wind Difficulty}_k = \begin{cases} 1 - [(x_1 - \text{wind}_k)/x_1], & \text{wind}_k < x_1 \\ 1 - [(0.5 - \text{wind}_k)/(0.5 - x1)], & x_1 \leq \text{wind}_k < 0.5 \\ 1 - [(x_2 - \text{wind}_k)/(x_2 - 0.5)], & 0.5 \leq \text{wind}_k < x_2 \\ 1 - [(\text{wind}_k - x_2)/(1 - x_2), & x2 \leq \text{wind}_k \end{cases} \tag{S5}$$

Essentially, we measure the percentage deviation from the most "ambiguous points" of each predictor value (humidity, temperature, wind) in a round. By subtracting the resulting value from 1, we have a higher value of Round Difficulty$_{i,k}$ indicating a more difficult round for participant *i*.

Majority Influence

Our approach to constructing majority influence is very similar to what we used to calculate minority influence.

We calculated the absolute difference between the median of the initial majority forecasts and the median final forecast for the other two sub-group sizes (2-person minority or 3-person mid-size sub-group):

$$\text{Majority Influence}^{\text{majority}}_{j,k} = 1 - |\text{med}(\text{prediction}^{\text{initial}}_{I^{\text{majority}}_{j,k}}) - \text{med}(\text{prediction}^{\text{final}}_{I^{\text{minority}}_{j,k}})| \tag{S6}$$

$$\text{Majority Influence}^{\text{mid-size}}_{j,k} = 1 - |\text{med}(\text{prediction}^{\text{initial}}_{I^{\text{majority}}_{j,k}}) - \text{med}(\text{prediction}^{\text{final}}_{I^{\text{mid-size}}_{j,k}})| \tag{S7}$$

Collective Error

Collective error was defined as the prediction error measured from the team median prediction excluding the participant's own prediction. The collective error for participant i of team j in round k is defined as:

$$\text{Collective Error}_{i,j,k} = |\text{med}(\text{prediction}^{\text{initial}}_{I^{-i}_j,k}) - \text{outcome}_k| \tag{S8}$$

Where the I^{-i}_j subscript indicates the participants in team j excluding participant i.

Training Error

In the accuracy regression models in the main text, we control for how well a participant learned the relationship between their assigned predictor and the outcome. We were interested in the participant's final learning at the end of the training phase. Therefore, we constructed training error as the average prediction error in the last half of the training phase, which corresponds to 55 rounds. We ignored rounds where no forecast was provided by a participant (we remove these observations from both the numerator and the denominator in the equation):

$$\text{training error}_i = \frac{\sum_{k=56}^{110} \text{prediction error}_{i,k}}{55} \tag{S9}$$

Supplementary tables

Table S1: Summary Statistics of Sample Demographics

	N	Mean	SD	Median	Min	Max
Age	295	31.837	10.083	30	18	66
Gender	292	0.524	0.500	1	0	1
n. of Submissions	299	178.930	189.366	114	1	1149
Approval Ratio	299	0.990	0.033	1	0.6	1
First Language	Percentage	Nationality	Percentage			
English	64.5	United Kingdom	38.8			
Other	22.1	Other 28.1				
Portuguese	7.0	United States	21.1			
Polish	6.4	Poland	6.4			
		Portugal	5.7			
Employment Status	Percentage	Student Status	Percentage			
Full-Time	47.8	No	66.6			
Other	23.4	Yes	30.8			
Part-Time	16.4	Other	2.7			
Unemployed (and job seeking)	12.4					

Table S2: Prediction Error by Algorithmic Players: Random *vs.* VAE (OLS regression)

	Prediction Error		
Predictors	Estimates	CI	*p-value*
(Intercept)	0.52***	0.47 – 0.58	<0.001
VAE	-0.06***	-0.08 – -0.03	<0.001
Avg. Difficulty	0.03	-0.05 – 0.11	0.495
Wind Minority	-0.03	-0.07 – 0.01	0.138
Humidity Minority	-0.06**	-0.11 – -0.02	0.003
Observations	1500		
R^2/R^2 adjusted	0.016 / 0.014		

Table S3: Summary of Mixed Model Estimates of Treatment Effect on full sample (full results including all predictors are reported in Tables S4-S7)

Dependent Variable:	*prediction error*		Δerror		Δdummy	
Predictors	Estimate	*p-value*	Estimate	*p-value*	Odds Ratio	*p-value*
(Intercept)	0.02	0.546	-0.09	0.129	0.98	0.866
Treatment	0.00	0.982	0.16'	*0.068*	1.41'	*0.081*
Temperature	-0.00	0.977	0.11	0.206	1.48*	**0.034**
Treatment : Temperature	0.00	0.95	-0.14	0.235	0.78	0.332
Wind	-0.06	0.142	0.15'	*0.08*	1.52*	**0.026**
Treatment : Wind	0.00	0.928	-0.26*	**0.033**	0.64'	*0.094*
Random effects	Yes		Yes		Yes	
Logistic Mixed Model	No		No		Yes	
Observations	14522		2536		2536	
Marginal / Conditional R^2	0.039 / 0.172		0.027 / 0.148		0.045 / 0.179	

Table S4: DV: prediction error, Δerror, Δdummy

Dependent Variable:	prediction error			Δerror			Δdummy		
Predictors	Estimates	CI	*p-value*	Estimates	CI	*p-value*	Odds Ratios	CI	*p-value*
(Intercept)	0.02	-0.04 – 0.08	0.546	-0.09	-0.22 – 0.03	0.129	0.98	0.75 – 1.28	0.866
Treatment	0.00	-0.08 – 0.08	0.982	0.16’	-0.01 – 0.33	0.068	1.41’	0.96 – 2.06	0.081
Temperature	-0.00	-0.08 – 0.07	0.977	0.11	-0.06 – 0.27	0.206	1.48*	1.03 – 2.13	0.034
Wind	-0.06	-0.14 – 0.02	0.142	0.15’	-0.02 – 0.32	0.08	1.52*	1.05 – 2.19	0.026
Round Difficulty	0.11***	0.09 – 0.13	<0.001	-0.04’	-0.08 – 0.00	0.057	0.96	0.88 – 1.05	0.413
Collective Error	0.06***	0.03 – 0.08	<0.001	-0.15***	-0.19 – -0.11	<0.001	0.68***	0.61 – 0.75	<0.001
Training Error	0.15***	0.13 – 0.17	<0.001	0.02	-0.03 – 0.07	0.525	0.93	0.84 – 1.04	0.228
Treatment x Temperature	0.00	-0.10 – 0.10	0.95	-0.14	-0.36 – 0.09	0.235	0.78	0.47 – 1.29	0.332
Treatment x Wind	0.00	-0.10 – 0.11	0.928	-0.26*	-0.50 – -0.02	0.033	0.64’	0.38 – 1.08	0.094
Random Effects									
σ^2	0.82			0.86			3.29		
τ_{00}	$0.12_{roundId}$			$0.10_{roundId}$			$0.44_{roundId}$		
	$0.02_{prolificId:gameId:date}$			$0.03_{prolificId:gameId:date}$			$0.09_{prolificId:gameId:date}$		
ICC	0.14			0.12			0.14		
N	$293_{prolificId}$			$244_{prolificId}$			$244_{prolificId}$		
	30_{gameId}			30_{gameId}			30_{gameId}		
	7_{date}			7_{date}			7_{date}		
	$1500_{roundId}$			$1290_{roundId}$			$1290_{roundId}$		
Observations	14522			2536			2536		
Marginal R2 / Conditional R2	0.039 / 0.172			0.027 / 0.148			0.045 / 0.179		

Table S5: DV: prediction error

Sample Dependent Variable:	Full prediction error			Temperature Subset prediction error			Humidity Subset prediction error			Wind Subset prediction error		
Predictors	Estimates	CI	*p-value*	Estimates	CI	*p-value*	Estimates	CI	*p-value*	Estimates	CI	*p-value*
(Intercept)	0.02	-0.04 – 0.08	0.546	0.02	-0.04 – 0.09	0.477	0.01	-0.05 – 0.08	0.74	-0.04	-0.10 – 0.02	0.155
Treatment	0.00	-0.08 – 0.08	0.982	0.00	-0.09 – 0.09	0.988	0.01	-0.08 – 0.10	0.868	0.01	-0.08 – 0.09	0.879
Round Difficulty	0.11***	0.09 – 0.13	<0.001	0.11***	0.07 – 0.14	<0.001	0.17***	0.13 – 0.20	<0.001	0.05**	0.02 – 0.09	0.002
Collective Error	0.06***	0.03 – 0.08	<0.001	0.02	-0.01 – 0.06	0.154	0.12***	0.08 – 0.15	<0.001	0.06***	0.03 – 0.09	<0.001
Training Error	0.15***	0.13 – 0.17	<0.001	0.12***	0.08 – 0.16	<0.001	0.11***	0.07 – 0.15	<0.001	0.21***	0.17 – 0.24	<0.001
Temperature	-0.00	-0.08 – 0.07	0.977									
Wind	-0.06	-0.14 – 0.02	0.142									
Treatment x Temperature	0.00	-0.10 – 0.10	0.95									
Treatment x Wind	0.00	-0.10 – 0.11	0.928									
Random Effects												
σ^2	0.82			0.75			0.49			0.72		
τ_{00}	$0.12_{roundId}$ $0.02_{prolificId:gameId:date}$			$0.26_{roundId}$ $0.02_{prolificId:gameId:date}$			$0.40_{roundId}$ $0.01_{prolificId:gameId:date}$			$0.17_{roundId}$ $0.01_{prolificId:gameId:date}$		
ICC	0.14			0.27			0.46			0.2		
N	$293_{prolificId}$ 30_{gameId} 7_{date} $1500_{roundId}$			$111_{prolificId}$ 30_{gameId} 7_{date} $1500_{roundId}$			$98_{prolificId}$ 30_{gameId} 7_{date} $1500_{roundId}$			$84_{prolificId}$ 30_{gameId} 7_{date} $1500_{roundId}$		
Observations	14522			5512			4826			4184		
Marginal R2 / Conditional R2	0.039 / 0.172			0.024 / 0.287			0.055 / 0.486			0.065 / 0.255		

Table S6: DV: Δerror

Sample	Full			Temperature Subset			Humidity Subset			Wind Subset		
Dependent Variable:	Δerror			Δerror			Δerror			Δerror		
Predictors	Estimates	CI	*p-value*	Estimates	CI	*p-value*	Estimates	CI	*p-value*	Estimates	CI	*p-value*
(Intercept)	-0.09	-0.22 – 0.03	0.129	0.01	-0.13 – 0.15	0.882	-0.10	-0.22 – 0.02	0.116	0.06	-0.03 – 0.15	0.217
Treatment	0.16’	-0.01 – 0.33	0.068	0.02	-0.17 – 0.21	0.831	0.19*	0.02 – 0.37	0.03	-0.09	-0.22 – 0.04	0.186
Round Difficulty	-0.04’	-0.08 – 0.00	0.057	-0.10**	-0.17 – -0.03	0.006	-0.01	-0.08 – 0.07	0.823	-0.00	-0.07 – 0.06	0.899
Collective Error	-0.15***	-0.19 – -0.11	<0.001	-0.19***	-0.26 – -0.12	<0.001	-0.06	-0.13 – 0.02	0.145	-0.14***	-0.20 – -0.08	<0.001
Training Error	0.02	-0.03 – 0.07	0.525	0.03	-0.08 – 0.14	0.581	0.05	-0.05 – 0.15	0.323	-0.00	-0.06 – 0.05	0.878
Temperature	0.11	-0.06 – 0.27	0.206									
Wind	0.15’	-0.02 – 0.32	0.08									
Treatment x Temperature	-0.14	-0.36 – 0.09	0.235									
Treatment x Wind	-0.26*	-0.50 – -0.02	0.033									
Random Effects												
σ^2	0.86			0.94			0.6			0.87		
τ_{00}	$0.10_{roundId}$ $0.03_{prolificId:gameId:date}$			$0.13_{roundId}$ $0.06_{prolificId:gameId:date}$			$0.32_{roundId}$ $0.02_{prolificId:gameId:date}$			$0.01_{roundId}$ $0.00_{prolificId:gameId:date}$		
ICC	0.12			0.16			0.36					
N	$244_{prolificId}$ 30_{gameId} 7_{date} $1290_{roundId}$			$87_{prolificId}$ 29_{gameId} 7_{date} $756_{roundId}$			$82_{prolificId}$ 30_{gameId} 7_{date} $582_{roundId}$			$75_{prolificId}$ 30_{gameId} 7_{date} $719_{roundId}$		
Observations	2536			972			727			837		
Marginal R2 / Conditional R2	0.027 / 0.148			0.038 / 0.195			0.015 / 0.372			0.025 / NA		

Table S7: DV: Δdummy

Sample	Full			Temperature Subset			Humidity Subset			Wind Subset		
Dependent Variable:	Δdummy			Δdummy			Δdummy			Δdummy		
Predictors	Odds Ratios	CI	*p-value*	Odds Ratios	CI	*p-value*	Odds Ratios	CI	*p-value*	Odds Ratios	CI	*p-value*
(Intercept)	0.98	0.75 – 1.28	0.866	1.35*	1.02 – 1.79	0.038	0.94	0.72 – 1.24	0.673	1.42**	1.16 – 1.76	0.001
Treatment	1.41'	0.96 – 2.06	0.081	1.14	0.78 – 1.66	0.505	1.44'	0.98 – 2.12	0.067	0.93	0.69 – 1.25	0.634
Round Difficulty	0.96	0.88 – 1.05	0.413	0.89	0.77 – 1.03	0.111	1.02	0.87 – 1.19	0.829	0.99	0.86 – 1.14	0.858
Collective Error	0.68***	0.61 – 0.75	<0.001	0.64***	0.55 – 0.74	<0.001	0.81*	0.68 – 0.96	0.014	0.71***	0.62 – 0.82	<0.001
Training Error	0.93	0.84 – 1.04	0.228	1.03	0.83 – 1.28	0.766	0.92	0.73 – 1.17	0.498	0.90	0.79 – 1.03	0.115
Temperature	1.48*	1.03 – 2.13	0.034									
Wind	1.52*	1.05 – 2.19	0.026									
Treatment x Temperature	0.78	0.47 – 1.29	0.332									
Treatment x Wind	0.64'	0.38 – 1.08	0.094									
**Random Effects**												
σ^2	3.29			3.29			3.29			3.29		
τ_{00}	$0.44_{roundId}$			$0.13_{roundId}$			$0.23_{roundId}$			$0.03_{roundId}$		
	$0.09_{prolificId:gameId:date}$			$0.18_{prolificId:gameId:date}$			$0.12_{prolificId:gameId:date}$			$0.01_{prolificId:gameId:date}$		
ICC	0.14			0.09			0.1			0.01		
N	$244_{prolificId}$			$87_{prolificId}$			$82_{prolificId}$			$75_{prolificId}$		
	30_{gameId}			29_{gameId}			30_{gameId}			30_{gameId}		
	7_{date}			7_{date}			7_{date}			7_{date}		
	$1290_{roundId}$			$756_{roundId}$			$582_{roundId}$			$719_{roundId}$		
Observations	2536			972			727			837		
Marginal R2 / Conditional R2	0.045 / 0.179			0.057 / 0.138			0.025 / 0.119			0.038 / 0.049		

Table S8: DV: median error, median error (bot), Δmedian error, Δmedian error (bot)

Dependent Variable:	median error			median error (bot)			Δmedian error			Δmedian error (bot)		
Predictors	Est.	CI	*p-value*	Est.	CI	*p-value*	Est.	CI	*p-value*	Est.	CI	*p-value*
(Intercept)	-0.10	-0.31 – 0.12	0.380	-0.10	-0.31 – 0.11	0.332	0.12	-0.07 – 0.32	0.212	0.09	-0.08 – 0.26	0.319
Treatment	0.24	-0.09 – 0.56	0.153	0.19	-0.13 – 0.51	0.249	-0.17	-0.46 – 0.13	0.266	-0.17	-0.42 – 0.09	0.205
Temp. Minority	0.18	-0.17 – 0.53	0.311	0.17	-0.17 – 0.51	0.326	-0.26	-0.57 – 0.05	0.096	-0.13	-0.41 – 0.14	0.348
Wind Minority	0.13	-0.16 – 0.41	0.374	0.15	-0.13 – 0.43	0.293	-0.25	-0.50 – 0.01	0.059	-0.12	-0.35 – 0.10	0.288
Round Difficulty	0.20	0.15 – 0.25	<0.001	0.18	0.14 – 0.23	<0.001	-0.14	-0.19 – -0.09	<0.001	-0.13	-0.17 – -0.10	<0.001
Training Error	0.13	0.03 – 0.23	0.012	0.11	0.01 – 0.21	0.026	-0.10	-0.19 – -0.01	0.033	-0.08	-0.16 – -0.01	0.035
Collective Error	0.20	0.15 – 0.24	<0.001	0.31	0.26 – 0.35	<0.001	0.41	0.35 – 0.46	<0.001	0.68	0.64 – 0.71	<0.001
Treatment x Temp. Minority	-0.49	-1.05 – 0.06	0.083	-0.38	-0.93 – 0.17	0.173	0.38	-0.12 – 0.88	0.133	0.28	-0.16 – 0.72	0.207
Treatment x Wind Minority	-0.29	-0.71 – 0.12	0.163	-0.24	-0.65 – 0.16	0.242	0.35	-0.02 – 0.72	0.067	0.22	-0.11 – 0.54	0.194
Random Effects												
σ^2	0.86			0.82			0.82			0.51		
τ_{00}	$0.04_{gameId:date}$			$0.04_{gameId:date}$			$0.03_{gameId:date}$			$0.03_{gameId:date}$		
ICC	0.05			0.05			0.03			0.05		
N	30_{gameId}			30_{gameId}			30_{gameId}			30_{gameId}		
	7_{date}			7_{date}			7_{date}			7_{date}		
Observations	1500			1500			1290			1500		
Marginal R2 / Conditional R2	0.109 / 0.150			0.150 / 0.189			0.165 / 0.193			0.470 / 0.495		

Table S9: Logit Mixed Model (DV: Δdummy) Estimates of Treatment Effect on Sample Subsets (w.r.t. minority). "O.R." stands for "Odds Ratios".

Sample	Full			Temperature Min.			Humidity Min.			Wind min.		
Dependent Variable:	Δdummy			Δdummy			Δdummy			Δdummy		
Predictors	O.R.	CI	*p-value*	O.R.	CI	*p-value*	O.R.	CI	*p-value*	O.R.	CI	*p-value*
(Intercept)	0.98	0.75 – 1.28	0.866	0.92	0.52 – 1.63	0.776	0.90	0.48 – 1.67	0.734	1.02	0.72 – 1.45	0.895
Treatment	1.41	0.96 – 2.06	0.081	2.63	1.15 – 6.00	0.022	1.70	0.63 – 4.61	0.294	1.12	0.69 – 1.82	0.637
Temperature	1.48	1.03 – 2.13	0.034	2.19	0.82 – 5.89	0.119	1.51	0.74 – 3.07	0.255	1.29	0.78 – 2.14	0.326
Wind	1.52	1.05 – 2.19	0.026	1.88	0.86 – 4.09	0.112	1.69	0.80 – 3.56	0.170	1.33	0.79 – 2.26	0.286
Round Difficulty	0.96	0.88 – 1.05	0.413	0.97	0.79 – 1.19	0.762	0.91	0.77 – 1.07	0.242	1.00	0.88 – 1.14	0.962
Training Error	0.93	0.84 – 1.04	0.228	0.92	0.64 – 1.30	0.626	0.98	0.78 – 1.22	0.823	0.92	0.80 – 1.07	0.298
Collective Error	0.68	0.61 – 0.75	<0.001	0.57	0.45 – 0.73	<0.001	0.62	0.52 – 0.75	<0.001	0.75	0.66 – 0.87	<0.001
Treatment x Temperature	0.78	0.47 – 1.29	0.332	0.29	0.09 – 0.95	0.041	0.59	0.19 – 1.82	0.357	1.18	0.60 – 2.32	0.623
Treatment x Wind	0.64	0.38 – 1.08	0.094	0.25	0.08 – 0.77	0.016	0.43	0.14 – 1.34	0.147	1.04	0.50 – 2.16	0.909
Random Effects												
σ^2	3.29			3.29			3.29			3.29		
τ_{00}	$0.44_{roundId}$<br>$0.09_{prolific\_id:gameID:date}$			$0.26_{roundId}$<br>$0.05_{prolific\_id:gameID:date}$			$0.33_{roundId}$<br>$0.05_{prolific\_id:gameID:date}$			$0.60_{roundId}$<br>$0.08_{prolific\_id:gameID:date}$		
ICC	0.14			0.09			0.10			0.17		
N	$244_{prolific\_id}$<br>30_{gameId}<br>7_{date}<br>$1290_{roundId}$			$41_{prolific\_id}$<br>5_{gameId}<br>5_{date}<br>$224_{roundId}$			$72_{prolific\_id}$<br>9_{gameId}<br>5_{date}<br>$372_{roundId}$			$131_{prolific\_id}$<br>16_{gameId}<br>7_{date}<br>$694_{roundId}$		
Observations	2536			466			747			1323		
Marginal R2 / Conditional R2	0.045 / 0.179			0.105 / 0.182			0.069 / 0.166			0.030 / 0.196		
